# Supplementary material for: Conditional cash transfers and mortality in people hospitalised with psychiatric disorders: A cohort study of the Brazilian Bolsa Família Programme
Source: PLoS Med. 2024 Dec 2;21(12):e1004486. doi: 10.1371/journal.pmed.1004486 (PMC11649113; doi:10.1371/journal.pmed.1004486)
Supplement: S1 Text — (DOCX) [file pmed.1004486.s002.docx]

**S1 Text. Accuracy analysis of the linkage between CadÚnico and the mortality information system in a randomized sample of 10 000 record pairs**

The data from the 100 million Brazilian Cohort was linked with records of the Bolsa Família Programme (BFP) payments, the Hospital Information System (SIH) and Mortality Information System (SIM). The linkage between the cohort and BFP utilized a deterministic approach, relying on a common correspondence key between the two databases (social identification number). Subsequently, record linkage using CIDACS-RL^1 2^, a tool for linking individual records in two stages using identifiers, was employed to connect information from the 100M Cohort (2001-2018) with SIH (2008-2018) and SIM (2000-2015). This process involved utilizing variables such as the name, mother's name, date of birth, sex, and municipality of residence^1 2^. The initial stage comprised deterministic linkage of five variables, followed by the second stage based on a similarity index derived from these variables^2^. To assess the accuracy of the linkage, a manual verification of a randomly selected sample was performed and evaluated through a receiver operating characteristic curve, considering sensitivity and specificity indexes (Figures 01 and 02). All linkage procedures were executed at the Center for Data and Knowledge Integration for Health (CIDACS)/ Fiocruz^3^, within a stringent data protection environment and in adherence to ethical and legal standards^4^.

**Summary of the databases used in the linkages:**

**Linkage between 100 million Brazilian Cohort and SIH:**

● Indexed database (the largest): 100 million cohort; period 2001-2018; number of

records 131,697,800.

● Search database (smaller database): SIH; period 2008-2018; number of records

27,858,929.

● Variables used: Name, mother’s name, date of birth, sex, and municipality of

residence.

● Number of linked records over the defined cut-off point: 7,802,044 (number of people hospitalised among those registered at CadÚnico)

**Linkage between 100 million Brazilian Cohort and SIM:**

● Indexed database (the largest): 100 million cohort; period 2001-2015; number of

records 114,008,317

● Search database (smaller database): SIM; period 2000-2015; number of records

17,829,111.

● Variables used: Name, Mother’s name, date of birth, sex, and municipality of

residence.

● Number of linked records over the defined cut-off point: 2,917,456 (number of people who died among those registered at CadÚnico)

References

1 Almeida D, Gorender D, Ichihara MY, Sena S, Menezes L, Barbosa GCG, et al. Examining the quality of record linkage process using nationwide Brazilian administrative databases to build a large birth cohort. *BMC Med Inform Decis Mak*. 2020;20(1):173. <https://pubmed.ncbi.nlm.nih.gov/32711532/>. [accessed: 15/05/2023]

2 Barbosa GCG, Ali MS, Araujo B, Reis S, Sena S, Ichihara MYT, et al. CIDACS-RL: a novel indexing search and scoring-based record linkage system for huge datasets with high accuracy and scalability. *BMC Med Inform Decis Mak*. 2020;20(289). <https://pubmed.ncbi.nlm.nih.gov/33167998/>. [accessed: 12/03/2023]

3 Barreto ML, Ichihara MY, Almeida BA, Barreto ME, Cabral L, Fiaccone RL, et al. The Center for Data and Knowledge Integration for Health (CIDACS): Linking health and social data in Brazil. *Int J Popul Data Sci*. 2019; 4(2):1-12. https://pubmed.ncbi.nlm.nih.gov/34095542/. [accessed: 02/02/2023]

4 Harron K, Dibben C, Boyd J, Hjern A, Azimaee M, Barreto ML, et al. Challenges in administrative data linkage for research. *Big Data Soc*. 2017;4(2):2053951717745678. <https://pubmed.ncbi.nlm.nih.gov/30381794/>. [accessed: 02/02/2023]
