# Supplementary material for: Conditional cash transfers and mortality in people hospitalised with psychiatric disorders: A cohort study of the Brazilian Bolsa Família Programme
Source: PLoS Med. 2024 Dec 2;21(12):e1004486. doi: 10.1371/journal.pmed.1004486 (PMC11649113; doi:10.1371/journal.pmed.1004486)
Supplement: S2 Text — (DOCX) [file pmed.1004486.s003.docx]

**S2 Text. Detailed information from eligible study population**

The eligible population for this study consisted of individuals from the 100 Million Cohor^1^ with a record of psychiatric hospitalisation, as either a primary or secondary diagnosis, between 2008 and 2015, when the complete data were available^2^. Initially, we identified 396,959 individuals hospitalised during this period. Next, we excluded 324,981 individuals who received the Bolsa Familia Programme (BFP) before hospitalisation to avoid selection bias. Beneficiaries might be less frequently hospitalised than non-beneficiaries, considering the association between reduction of poverty and improved health conditions^3 4 5^, causing an imbalance with the comparison group. We then excluded 500 individuals (<1%) under 10 years old, considering that suicide is rare in this population^6^, as well as those over 120 years old. We also excluded 410 individuals (<1%) with repeated hospitalisations during the period to enable greater comparability between subjects since multiple hospitalisations could be associated with a higher severity of diagnosis^7^. Finally, we excluded 1,167 individuals (<1%) due to data inconsistencies related with discrepancies between dates of death (n=196), registration (n=889), and receipt of BFP (n=82), which may reflect linkage errors. Therefore, the study population consisted of 69,901 individuals.

References

1 Barreto ML, Ichiara MY, Pescarini JM, Ali MS, Borges GL, Fiaccone RL, et al. Cohort Profile: The 100 Million Brazilian Cohort. *Int J Epidemiol*. 2022; 51(2): e27-e38. https://pubmed.ncbi.nlm.nih.gov/34922344/. [accessed: 02/02/2023]

2 Machado DB, Azevedo JPA, Alves FJO, Castro-de-Araujo LFS, Silva ER, Fialho EMX, et al. The impact of social drivers, conditional cash transfers and their mechanisms on the mental health of the young; an integrated retrospective and forecasting approach using the 100 million Brazilian Cohort: A study protocol. *PLoS One*.2022; 17(10): e0272481. https://pubmed.ncbi.nlm.nih.gov/36201469/. [accessed: 02/02/2023]

3 Alves FJO, Ramos D, Paixão ES, Falcão IR, de Cássia Ribeiro-Silva R, Fiaccone R, et al. Association of Conditional Cash Transfers With Maternal Mortality Using the 100 Million Brazilian Cohort. *JAMA Netw Open*. 2023; 6(2):e230070. https://pubmed.ncbi.nlm.nih.gov/36821115/. [accessed: 11/03/2023]

4 Jesus GS, Pescarini JM, Silva AF, Torrens A, Carvalho WM, Junior EPP et al. The effect of primary health care on tuberculosis in a nationwide cohort of 7·3 million Brazilian people: a quasi-experimental study. *Lancet Glob Health*. 2022; 10(3): e390-e397 <https://pubmed.ncbi.nlm.nih.gov/35085514/>. [accessed: 20/09/2023]

5 Ramos D, da Silva NB, Ichihara MY, Fiaccone RL, Almeida D, Sena S et al. Conditional cash transfer program and child mortality: A cross-sectional analysis nested within the 100 Million Brazilian Cohort. *PLoS Med*. 2021; 18(9): e1003509. https://pubmed.ncbi.nlm.nih.gov/34582433/. [accessed: 12/03/2023]

6 Machado DB, Williamson E, Pescarini JM, Alves FJO, Castro-de-Araujo LFS, Ichihara MY, et al. Relationship between the Bolsa Família national cash transfer programme and suicide incidence in Brazil: A quasi-experimental study. *PLoS Med*. 2022; 19(5): e1004000. https://pubmed.ncbi.nlm.nih.gov/35584178/. [accessed: 02/02/2023]

7 Mascayano F, Haselden M, Corbeil T, Wall MM, Tang F, Essock SM et al. Patient-, Hospital-, and System-Level Factors Associated With 30-Day Readmission After a Psychiatric Hospitalization. *J Nerv Ment Dis*. 2022; 210(10): 741-746. https://pubmed.ncbi.nlm.nih.gov/35472041/. [accessed: 15/05/2023]
