# Supplementary material for: Conditional cash transfers and mortality in people hospitalised with psychiatric disorders: A cohort study of the Brazilian Bolsa Família Programme
Source: PLoS Med. 2024 Dec 2;21(12):e1004486. doi: 10.1371/journal.pmed.1004486 (PMC11649113; doi:10.1371/journal.pmed.1004486)
Supplement: S3 Text — (DOCX) [file pmed.1004486.s004.docx]

**S3 Text. Propensity score: definition, estimation, summary, and support graphs**

PS is employed when comparing intervention and non-intervention groups, as the allocation of these groups is not randomized^1^. This lack of randomization can result in imbalanced distribution of covariates, leading to biased estimates^2^. By controlling for confounding factors, PS aids in correcting the estimation of the intervention’s effect^2^. The propensity scores were obtained from a logistic regression^2^ to estimate the conditional probability of receiving BFP (S4 Table 2). Then, we assessed the common support graph (S4 Fig.3) and we compared the range of propensity scores among BFP and non-BFP groups. A summary of the propensity score stratified by BFP groups was shown in S4 Table 3. After estimating the PS for receiving BFP from the cohort baseline sociodemographic covariates, we calculated the weights for BFP beneficiary families (weight = 1) and non-BFP beneficiary families (weight = PS/(1 - PS)) using inverse probability of treatment weighting (IPTW). We estimated the Cox regression using IPTW and compared the differences in the distribution of PS covariates between beneficiaries and non-beneficiaries using covbal command from Stata, to assess the balance of potential confounders before and after IPTW weighting.

References

1 Williamson E, Morley R, Lucas A, Carpenter J. Propensity scores: from naive enthusiasm to intuitive understanding. *Stat Methods Med Res*.2012; 21(3):273-293. https://pubmed.ncbi.nlm.nih.gov/21262780/. [accessed: 06/11/2023]

2 Ali MS, Prieto-Alhambra D, Lopes LC, Ramos D, Bispo N, Ichihara MY, et al. Propensity Score Methods in Health Technology Assessment: Principles, Extended Applications, and Recent Advances. *Front Pharmacol*. 2019; 10(973): 1-19. https://pubmed.ncbi.nlm.nih.gov/31619986/. [accessed: 20/07/2024]
