# Supplementary material for: Conditional cash transfers and mortality in people hospitalised with psychiatric disorders: A cohort study of the Brazilian Bolsa Família Programme
Source: PLoS Med. 2024 Dec 2;21(12):e1004486. doi: 10.1371/journal.pmed.1004486 (PMC11649113; doi:10.1371/journal.pmed.1004486)
Supplement: S4 Text — (DOCX) [file pmed.1004486.s005.docx]

**S4 Text. Intraclass correlation coefficient estimation**

Although the BFP information is at the individual level, we calculated the intraclass correlation coefficient (ICC) to check for a possible influence of the household level, as this benefit is received by the family as well as to assess the need to adjust the model considering this influence. To this end, we ran an adjusted mixed-effects Poisson model with fixed effects considering household identification as the cluster and overall mortality as the outcome (S6 Table 12a), and subsequently, the ICC was estimated following previously published recommendations^1^. The results indicated a low correlation between the household and individual levels (S6 Table 12b) as well as a null association between BFP and overall mortality when household level was accounted for (S6 Table 12a). The number of household members hospitalised with psychiatric disorders ranged from 1 to 4. The analysis included 69,192 households, with household sizes varying between 1 and 40 members (S6 table 12b).

Reference

1 Karry SM, Bland AM. The intraclass correlation coefficient in cluster randomisation. *BMJ.* 1998; 316: 1455. <https://www.bmj.com/content/316/7142/1455.1>. [accessed: 20/07/2024]
