# Supplementary material for: Conditional cash transfers and mortality in people hospitalised with psychiatric disorders: A cohort study of the Brazilian Bolsa Família Programme
Source: PLoS Med. 2024 Dec 2;21(12):e1004486. doi: 10.1371/journal.pmed.1004486 (PMC11649113; doi:10.1371/journal.pmed.1004486)
Supplement: S5 Text — (DOCX) [file pmed.1004486.s006.docx]

**S5 Text. Description of individuals excluded from the analysis**

A descriptive analysis of the individuals excluded from the analysis is provided below. We excluded individuals who were registered in CadÚnico before hospitalisation, using the same eligibility criteria as in the main analysis.
