# Supplementary material for: Conditional cash transfers and mortality in people hospitalised with psychiatric disorders: A cohort study of the Brazilian Bolsa Família Programme
Source: PLoS Med. 2024 Dec 2;21(12):e1004486. doi: 10.1371/journal.pmed.1004486 (PMC11649113; doi:10.1371/journal.pmed.1004486)
Supplement: S6 Text — (DOCX) [file pmed.1004486.s007.docx]

**S6 Text. Summary of the dataset’s description**

**100 million Brazilian Cohort baseline**

The 100 Million Brazilian Cohort baseline is a dataset developed by Center for Data and Knowledge Integration for Health (CIDACS / FIOCRUZ) to investigate social determinants and the impact of social programs and policies on various health contexts in Brazil^1^. This dataset is based on information from over 131 million individuals who were registered between 2001 and 2018 in CadÚnico, the primary system for applying for social benefits in Brazil which include the Bolsa Família Programme (BFP)^1 2^. It includes socio-economic and sociodemographic information from poorer Brazilian individuals and their families who apply for social programs such as BFP^2^. To qualify and register with CadÚnico, families must have a per capita income of up to half a minimum wage or a total family income of up to three minimum wages^2^. Although the 100 Million Brazilian Cohort baseline dataset has been used in several studies by CIDACS^3 4 5 6 7^, this is the first time that we have focused on a specific vulnerable population—those hospitalised due to psychiatric disorders within this large cohort.

**Hospitalisation Information System (SIH)**

The SIH is the national dataset for all hospitalizations funded by the Brazilian public health system, including both general and specialized hospitals^3^. Although Brazil also has a private health system, around 75% of the Brazilian population relies on the public system^8^. The SIH was filled out by health professionals who use standardised forms according to updated International Classification of Disease (ICD-10) to record primary and secondary causes of a hospital admission^8^. In this study, we used only information on hospitalizations due to psychiatric disorders.

**Mortality Information System (SIM)**

The SIM is a dataset responsible for the mandatory registration of all deaths in Brazil^3^. This system is also filled out by health professionals using standardized forms based on the updated ICD-10 for all causes of death^3^. The SIM has been recognized for its high quality and completeness^9^.

References

1 Barreto ML, Ichihara MY, Almeida BA, Barreto ME, Cabral L, Fiaccone RL, et al. The Center for Data and Knowledge Integration for Health (CIDACS): Linking health and social data in Brazil. *Int J Popul Data Sci*. 2019; 4(2):1-12. https://pubmed.ncbi.nlm.nih.gov/34095542/. [accessed: 02/02/2023]

2 Barreto ML, Ichiara MY, Pescarini JM, Ali MS, Borges GL, Fiaccone RL, et al. Cohort Profile: The 100 Million Brazilian Cohort. *Int J Epidemiol*. 2022; 51(2): e27-e38. https://pubmed.ncbi.nlm.nih.gov/34922344/. [accessed: 02/02/2023]

3 Machado DB, Azevedo JPA, Alves FJO, Castro-de-Araujo LFS, Silva ER, Fialho EMX, et al. The impact of social drivers, conditional cash transfers and their mechanisms on the mental health of the young; an integrated retrospective and forecasting approach using the 100 million Brazilian Cohort: A study protocol. *PLoS One*.2022; 17(10): e0272481. https://pubmed.ncbi.nlm.nih.gov/36201469/. [accessed: 02/02/2023]

4 Alves FJO, Ramos D, Paixão ES, Falcão IR, de Cássia Ribeiro-Silva R, Fiaccone R, et al. Association of Conditional Cash Transfers With Maternal Mortality Using the 100 Million Brazilian Cohort. *JAMA Netw Open*. 2023; 6(2):e230070. https://pubmed.ncbi.nlm.nih.gov/36821115/. [accessed: 11/03/2023]

5 Jesus GS, Pescarini JM, Silva AF, Torrens A, Carvalho WM, Junior EPP et al. The effect of primary health care on tuberculosis in a nationwide cohort of 7·3 million Brazilian people: a quasi-experimental study. *Lancet Glob Health*. 2022; 10(3): e390-e397 <https://pubmed.ncbi.nlm.nih.gov/35085514/>. [accessed: 20/09/2023]

6 Ramos D, da Silva NB, Ichihara MY, Fiaccone RL, Almeida D, Sena S et al. Conditional cash transfer program and child mortality: A cross-sectional analysis nested within the 100 Million Brazilian Cohort. *PLoS Med*. 2021; 18(9): e1003509. https://pubmed.ncbi.nlm.nih.gov/34582433/. [accessed: 12/03/2023]

7 Machado DB, Williamson E, Pescarini JM, Alves FJO, Castro-de-Araujo LFS, Ichihara MY, et al. Relationship between the Bolsa Família national cash transfer programme and suicide incidence in Brazil: A quasi-experimental study. *PLoS Med*. 2022; 19(5): e1004000. https://pubmed.ncbi.nlm.nih.gov/35584178/. [accessed: 02/02/2023]

8 Cerqueira DRC, Alves PP, Coelho DCS, Reis MVM, Lima AS. Uma análise da base de dados do Sistema de Informação Hospitalar entre 2001 e 2018: dicionário dinâmico, disponibilidade dos dados e aspectos metodológicos para a produção de indicadores sobre violência. Rio de Janeiro; IPEA, 2019. https://repositorio.ipea.gov.br/handle/11058/9409. [accessed: 20/09/2023]

9 World Health Organization (WHO). WHO methods and data sources for country-level causes of death 2000-2019. Geneva: WHO; 2020. Available at: https://platform.who.int/mortality/about/data-quality. [accessed: 20/09/2023]
