# Supplementary figures and images for: Conditional cash transfers and mortality in people hospitalised with psychiatric disorders: A cohort study of the Brazilian Bolsa Família Programme
Source: PLoS Med. 2024 Dec 2;21(12):e1004486. doi: 10.1371/journal.pmed.1004486 (PMC11649113; doi:10.1371/journal.pmed.1004486)

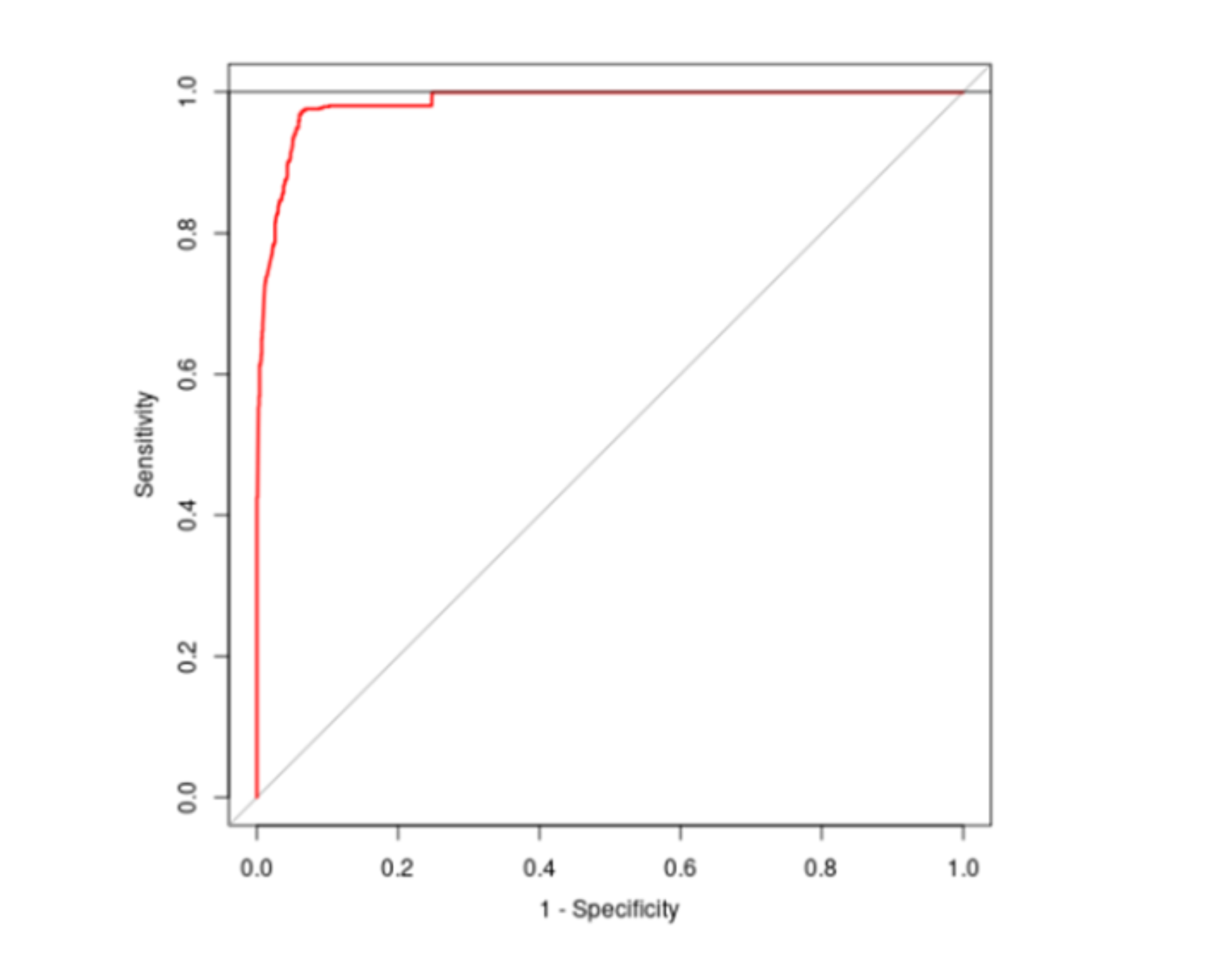

Supplement: S1 Fig — Source: Developed by the CIDACS Data Production Center. (TIF) [file pmed.1004486.s008.tif]

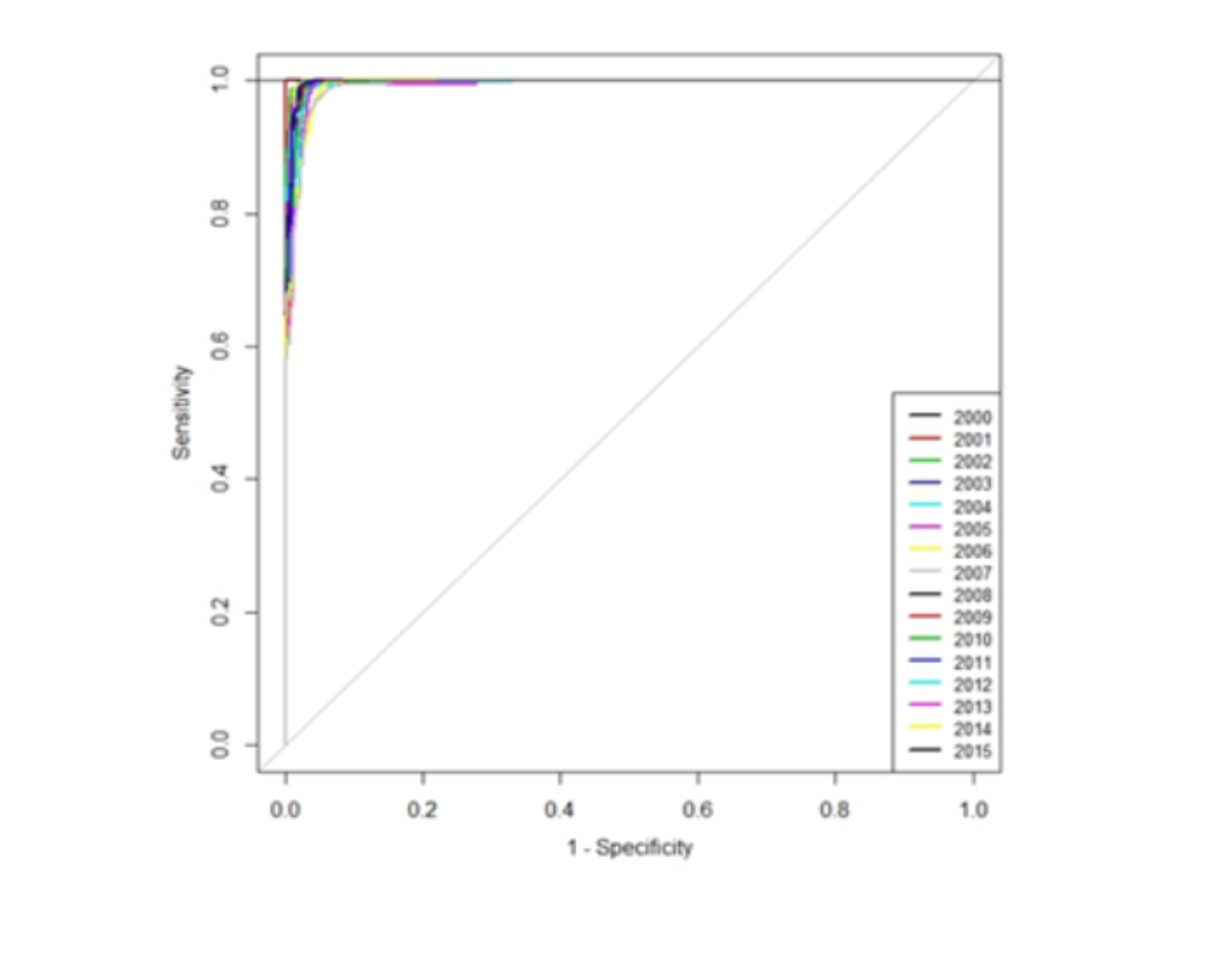

Supplement: S2 Fig — Source: Developed by the CIDACS Data Production Center. (TIF) [file pmed.1004486.s009.tif]

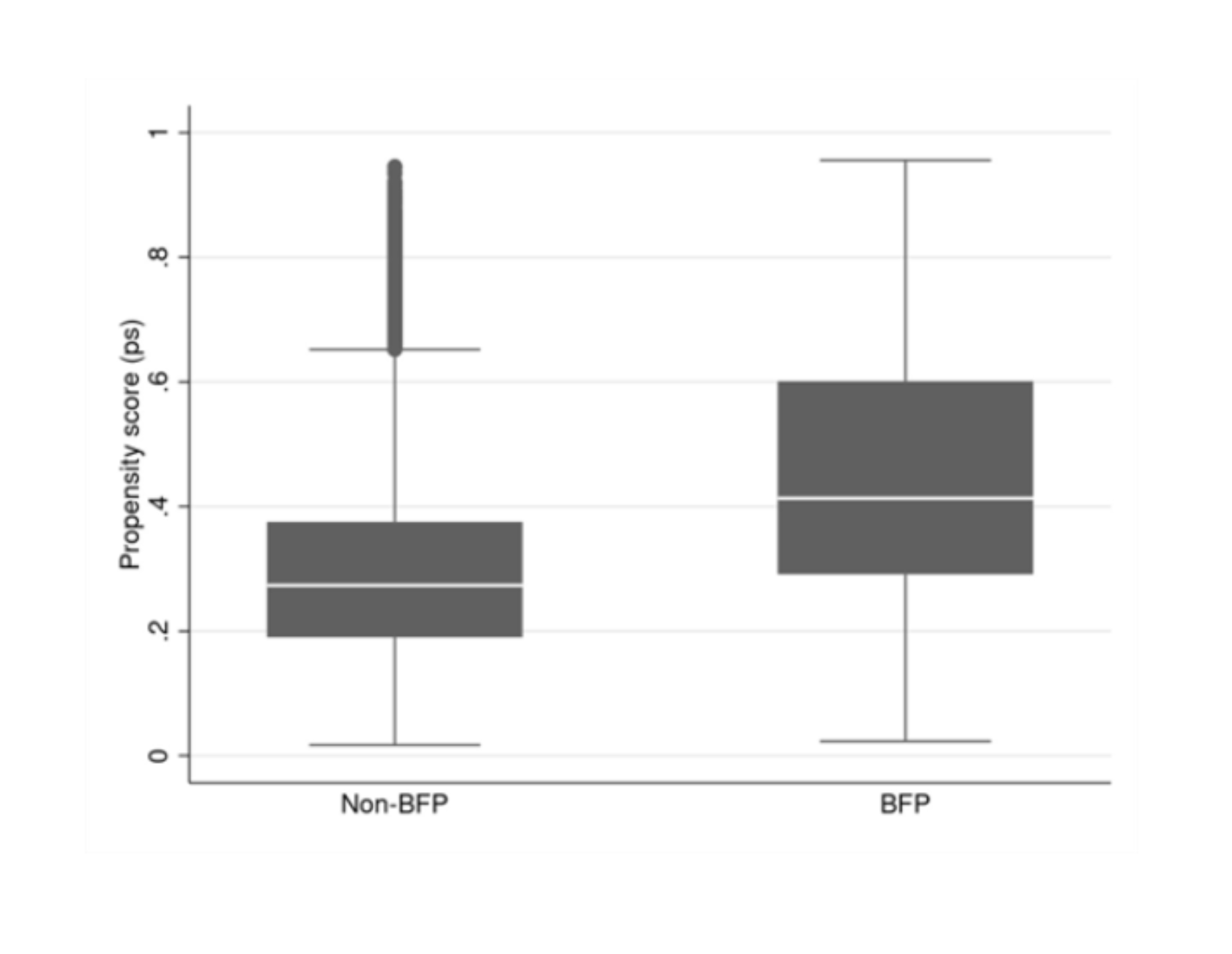

Supplement: S3 Fig — (TIF) [file pmed.1004486.s010.tif]
