# Supplementary material for: Conditional cash transfers and mortality in people hospitalised with psychiatric disorders: A cohort study of the Brazilian Bolsa Família Programme
Source: PLoS Med. 2024 Dec 2;21(12):e1004486. doi: 10.1371/journal.pmed.1004486 (PMC11649113; doi:10.1371/journal.pmed.1004486)
Supplement: S1 Table — (DOCX) [file pmed.1004486.s011.docx]

**S1 Table.** Mortality rates overall and by subgroups through receipt of the Bolsa Familia Programme, 2008-2015.

|  | **BFP (n=26,556)** | | | **Non-BFP (n=43,345)** | | |
| --- | --- | --- | --- | --- | --- | --- |
|  | **Events** | **Person-years at risk** | **Incidence rate^1^**  **(95% CI)** | **Events** | **Incidence rate^1^**  **(95% CI)** | **Person-years at risk** |
| **Overall mortality** | 2,960 | 83590.94 | 3541.05  (3415.76, 3670.94) | 5,158 | 4816.64  (4686.97, 4949.89) | 107087.21 |
| Male  Female | 2,218  742 | 45589.60  38001.37 | 4865.14  (4666.83, 5071.89)  1952.57  (1817.01, 2098.23) | 3,655  1,503 | 5443.56  (5269.92, 5622.93)  3762.79  (3577.29, 3957.92) | 67143.49  39943.71 |
| 10 -24  25 - 59  > 60 | 131  2,538  291 | 11050.30  69984.54  2556.09 | 1185.49  (998.91, 1406.91)  3626.51  (3488.14, 3770.38)  11384.59  (10148.9, 12770.73) | 127  3,544  1,487 | 1546.89  (1299.95, 1840.73)  4074.04  (3942.10, 4210.40)  12509.05  (11889.14, 13161.28) | 8210.04  86989.77  11887.395 |
| **Natural causes** | 2,327 | 83590.94 | 2783.79  (2672.95, 2899.23) | 4,367 | 4077.98  (3958.81, 4200.74) | 107087.21 |
| Male  Female | 1,711  616 | 45589.60  38001.34 | 3753.05  (3579.36, 3935.16)  1620.99  (1497.91, 1754.19) | 3,018  1,349 | 4494.85  (4337.31, 4658.10)  3377.25  (3201.76, 3562.37) | 67143.49  39943.71 |
| 10 -24  25 - 59  > 60 | 46  2,007  274 | 11050.30  69984.54  2556.09 | 416.28  (311.80, 555.76)  2867.78  (2745.02, 2996.02)  10719.50  (9522.52, 12066.96) | 62  2,895  1,410 | 755.17 (588.76,968.61)  3327.98  (3208.93, 3451.44)  11861.30  (9522.52, 12066.96) | 8210.04  86989.77  11887.395 |
| **Unnatural causes** | 633 | 83590.94 | 757.26  (700.51, 818.61) | 791 | 738.65  (688.93, 791.96) | 107087.21 |
| Male  Female | 507  126 | 45589.60  38001.34 | 1112.10  (1019.39, 1213.24)  331.57  (278.45, 394.82) | 637  154 | 948.71  (877.82, 1025.32)  385.54 (329.22 451.51) | 67143.49  39943.71 |
| 10 -24  25 - 59  > 60 | 85  531  17 | 11050.30  69984.54  2556.09 | 769.21  (621.90, 951.42)  758.74  (696.87, 826.10)  665.08  (413.45, 1069.84) | 65  649  77 | 791.71  (620.85, 1009.59)  746.06  (690.81, 805.73)  647.74  (518.08, 809.85) | 8210.04  86989.77  11887.395 |
| **Suicide** | 121 | 83590.94 | 144.75  (121.13, 172.98) | 189 | 176.49  (153.04, 203.53) | 107087.21 |
| Male  Female | 84  37 | 45589.60  38001.34 | 184.25  (148.78, 228.18)  97.36  (70.54, 134.38) | 146  43 | 217.44  (184.88, 255.74)  107.65  (79.84, 145.15) | 67143.49  39943.71 |
| 10 -24  25 - 59  > 60 | 12  108  01 | 11050.30  69984.54  2556.09 | 108.59  (61.67, 191.22)  154.32  (127.79, 186.34)  39.12  (5.51, 277.73) | 13  153  23 | 158.34  (91.94, 272.69)  175.88  (150.11, 206.08)  193.48  (128.57, 291.16) | 8210.04  86989.77  11887.395 |

^1^ Estimated rates per 100,000.
