# Supplementary material for: Conditional cash transfers and mortality in people hospitalised with psychiatric disorders: A cohort study of the Brazilian Bolsa Família Programme
Source: PLoS Med. 2024 Dec 2;21(12):e1004486. doi: 10.1371/journal.pmed.1004486 (PMC11649113; doi:10.1371/journal.pmed.1004486)
Supplement: S2 Table — (DOCX) [file pmed.1004486.s012.docx]

**S2A Table**. Logistic regression to estimate propensity scores for receiving Bolsa Familia according to covariables, N= 57,905

| **Variable** | **Odds Ratio**  **(95% CI)** | **p value** |
| --- | --- | --- |
| Sex  Male  Female | 1.00  1.60 (1.54,1.66) | <0.001 |
| Age group (years old)  10-24  25-59  >60 | 1.00  0.61 (0.57,0.64)  0.20 (0.18, 0.22) | <0.001  <0.001 |
| Education Level (years of education)  Never been study  Preschool  Primary school or less (<= 5 years)  Junior high school (6- 10 years)  High school (10-12 years)  College/university (>=13 years) | 1.00  0.95 (0.78,1.15)  1.66 (1.56,1.77)  2.01 (1.88,2.15)  1.79 (1.67,1.91)  1.08 (0.93,1.26) | 0.591  <0.001  <0.001  <0.001  0.314 |
| Race  White  Black  Asian  Brown  Indigenous | 1.00  1.32 (1.22,1.41)  1.12 (0.86,1.46)  1.20 (1.15,1.25)  3.09 (1.87,5.07) | <0.001  0.407  <0.001  <0.001 |
| Location of residence  Urban  Rural | 1.00  1.03 (0.94,1.12) | 0.572 |
| Brazilian regions  Southeast  Northeast  Midwest  South  North | 1.00  0.85 (0.80,0.90)  0.59 (0.54,0.63)  0.74 (0.70,0.78)  0.69 (0.57,0.78) | <0.001  <0.001  <0.001  <0.001 |
| Household characteristics  Water supply  Public Network (running water)  Well, natural sources, or other  Waste  Public collection system  Burned, buried, outdoor disposal, o other  Sanitation  Public network  Septic tank  Homemade septic tank  Ditch or other  Construction materials  Bricks/ cement  Wood, other vegetal materials, and other | 1.00  1.44 (1.35,1.55)  1.00  0.97 (0.88,1.07)  1.00  1.02 (0.96,1.08)  0.98 (0.93,1.04)  1.45 (1.30,1.62)  1.00  1.16 (1.09,1.23) | <0.001  0.578  0.592  0.599  <0.001  <0.001 |
| Isolation  Live with someone else  Live alone | 1.00  1.23 (1.17,1.28) | <0.001 |
| Year of registration at CadÚnico  2008  2009  2010  2011  2012  2013  2014  2015 | 1.00  1.02 (0.86,1.21)  0.69 (0.59,0.81)  0.23 (0.19,0.27)  0.15 (0.13,0.18)  0.18 (0.16,0.21)  0.14 (0.12,0.16)  0.10 (0.09,0.11) | 0.794  <0.001  <0.001  <0.001  <0.001  <0.001  <0.001 |

**S2B Table**. Propensity score description in accordance with the confounding covariates observed, Brazil, 2008 to 2015, N= 57,905

| **Propensity score** | **BFP**  **N= 20,399** | **Non- BFP**  **N= 37,506** |
| --- | --- | --- |
| Average | 0.442 | 0.303 |
| Deviation population | 0.197 | 0.144 |
| Median | 0.394 | 0.283 |
| Minimum | 0.038 | 0.002 |
| Maximum | 0.960 | 0.936 |
| 25^th^ Percentile | 0.290 | 0.208 |
| 75^th^ Percentile | 0.595 | 0.372 |
