# Supplementary material for: Conditional cash transfers and mortality in people hospitalised with psychiatric disorders: A cohort study of the Brazilian Bolsa Família Programme
Source: PLoS Med. 2024 Dec 2;21(12):e1004486. doi: 10.1371/journal.pmed.1004486 (PMC11649113; doi:10.1371/journal.pmed.1004486)
Supplement: S3 Table — (A) ATT of overall mortality for BFP receipt between 2008 and 2015 using KM. (B) ATT of natural causes of death for BFP receipt between 2008 and 2015 using KM. (C) ATT of unnatural causes of death for BFP receipt between 2008 and 2015 using KM. (D) ATT of suicide for BFP receipt between 2008 and 2015 using KM. (DOCX) [file pmed.1004486.s013.docx]

**S3A Table**. ATT of overall mortality for BFP receipt between 2008 and 2015 using Kernel matching

|  | Kernel Weighting  ATT* (95% CI) |
| --- | --- |
| ATT | 0.01 (0.007, 0.018) |
| N | 57,905 |

* *Average treatment effect on the treated (ATT) estimated using kernel matching (PS variables).

**S3B Table**. ATT of natural causes of death for BFP receipt between 2008 and 2015 using Kernel matching

|  | Kernel Weighting  ATT* (95% CI) |
| --- | --- |
| ATT | 0.00 (0.001, 0.011) |
| N | 57,905 |

* *Average treatment effect on the treated (ATT) estimated using kernel matching (PS variables).

**S3C Table**. ATT of unnatural causes of death for BFP receipt between 2008 and 2015 using Kernel matching

|  | Kernel Weighting  ATT* (95% CI) |
| --- | --- |
| ATT | 0.00 (0.003, 0.008) |
| N | 57,905 |

* *Average treatment effect on the treated (ATT) estimated using kernel matching (PS variables).

**S3D Table**. ATT of suicide for BFP receipt between 2008 and 2015 using Kernel matching

|  | Kernel Weighting  ATT* (95% CI) |
| --- | --- |
| ATT | 0.00 (-0.004, 0.019) |
| N | 57,905 |

* *Average treatment effect on the treated (ATT) estimated using kernel matching (PS variables).
