# Supplementary material for: Conditional cash transfers and mortality in people hospitalised with psychiatric disorders: A cohort study of the Brazilian Bolsa Família Programme
Source: PLoS Med. 2024 Dec 2;21(12):e1004486. doi: 10.1371/journal.pmed.1004486 (PMC11649113; doi:10.1371/journal.pmed.1004486)
Supplement: S4 Table — (DOCX) [file pmed.1004486.s014.docx]

**S4 Table.** Crude and adjusted association of Bolsa Família Programme participation with overall, natural, unnatural, and suicide mortalities, 2008-2015.

|  |  | **Cox Model** | **Competitive risk model** | | |
| --- | --- | --- | --- | --- | --- |
|  |  | **Overall mortality** | **Natural causes** | **Unnatural causes** | **Suicide** |
| **Confounder adjustment** | **Overall population** | **IRR (95% CI)** | **IRR (95% CI)** | **IRR (95% CI)** | **IRR (95% CI)** |
| Cox with no adjustment  Non-BFP  BFP  p value | 69,901 | 1.00  0.67 (0.64, 0.70)  <0.001 | 1.00  0.62 (0.59, 0.65)  <0.001 | 1.00  1.08 (0.96, 1.22)  0.204 | 1.00  0.84 (0.66, 1.05)  0.121 |
| Cox with adjustment^1^  Non-BFP  BFP  p value | 57,905 | 1.00  1.05 (0.99 – 1.12)  0.061 | 1.00  1.04 (0.98, 1.11)  0.180 | 1.00  1.15 (0.99, 1.34)  0.056 | 1.00  0.93 (0.70, 1.22)  0.590 |

Abbreviations: BFP - Bolsa Família Programme; HR - Hazard Ratio; CI - confidence interval.

1 HR adjusted for sex, age, race, education level, household characteristics (water supply, waste, sanitation, and construction materials), living alone, crowding, Brazilian region, location of residence, length and year of hospitalisation, and year of CadÚnico registration.
