# Supplementary material for: Conditional cash transfers and mortality in people hospitalised with psychiatric disorders: A cohort study of the Brazilian Bolsa Família Programme
Source: PLoS Med. 2024 Dec 2;21(12):e1004486. doi: 10.1371/journal.pmed.1004486 (PMC11649113; doi:10.1371/journal.pmed.1004486)
Supplement: S5 Table — (DOCX) [file pmed.1004486.s015.docx]

**S5 Table**. Association of Bolsa Família Programme participation with overall, natural, unnatural, and suicide mortalities accounting for missing data, 2008-2015.

|  |  | **Cox Model** | **Competitive risk model** | | |
| --- | --- | --- | --- | --- | --- |
|  |  | **Overall mortality** | **Natural causes** | **Unnatural causes** | **Suicide** |
| **Confounder adjustment** | **Overall population** | **IRR (95% CI)** | **IRR (95% CI)** | **IRR (95% CI)** | **IRR (95% CI)** |
| Cox adjusted with IPTW^1^ (final model)  Non-BFP  BFP  p value | 69,901 | 1.00  0.93 (0.88, 0.99)  0.041 | 1.00  0.90 (0.84, 0.97)  0.004 | 1.00  1.14 (0.98, 1.35)  0.090 | 1.00  0.91 (0.68 – 1.22)  0.534 |

Abbreviations: BFP - Bolsa Família Programme; HR - Hazard Ratio; CI - confidence interval.

1 HR estimated with IPTW given sex, age, race, education level, household characteristics (water supply, waste, sanitation, and construction materials), living alone, crowding, Brazilian region, location of residence, length and year of hospitalisation, and year of CadÚnico registration.
