# Supplementary material for: Conditional cash transfers and mortality in people hospitalised with psychiatric disorders: A cohort study of the Brazilian Bolsa Família Programme
Source: PLoS Med. 2024 Dec 2;21(12):e1004486. doi: 10.1371/journal.pmed.1004486 (PMC11649113; doi:10.1371/journal.pmed.1004486)
Supplement: S7 Table — (A) Intraclass correlation estimation for the household level. (B) Association of BFP participation with overall mortality considering household level, 2008–2015. (DOCX) [file pmed.1004486.s017.docx]

**S7A Table. Association of Bolsa Família Programme participation with overall mortality considering household level, 2008-2015.**

| **Confounder adjustment** | **Overall population** | **IRR (95% CI)** |
| --- | --- | --- |
| Poisson mixed effect adjusted with IPTW^1^  Non-BFP  BFP  p value | 57,905 | 1.00  0.98  (0.92 – 1.04)  0.478 |

Abbreviations: BFP - Bolsa Família Programme; IRR - incidence rate ratio; CI - confidence interval; IPTW - inverse probability of treatment weighting.

1 IRR estimated with IPTW given sex, age, race, education level, household characteristics (water supply, waste, sanitation, and construction materials), living alone, crowding, Brazilian region, location of residence, length and year of hospitalisation, and year of CadÚnico registration.

**S7B Table. Intraclass correlation estimation for the household level**

| Variable | ICC | Standard error | 95% CI |
| --- | --- | --- | --- |
| Household level | 0.016 | 0.381 | 0.001, 0.620 |

Abbreviation: ICC, Intraclass correlation estimation. CI, Confidence Interval
