# Supplementary material for: Conditional cash transfers and mortality in people hospitalised with psychiatric disorders: A cohort study of the Brazilian Bolsa Família Programme
Source: PLoS Med. 2024 Dec 2;21(12):e1004486. doi: 10.1371/journal.pmed.1004486 (PMC11649113; doi:10.1371/journal.pmed.1004486)
Supplement: S8 Table — (DOCX) [file pmed.1004486.s018.docx]

**S8 Table.** Description of individuals excluded from the analysis following definition of Bolsa Familia Programme exposition, 2008-2015.

|  | **Participants, No. (%)** | | |
| --- | --- | --- | --- |
| **Characteristics** | **Overall**  **N=308,354** | **BFP**  **N=254,589 (82.6%)** | **Non-BFP**  **N= 53,765 (17.5%)** |
|  | N (%) or mean (SD) | N (%) or mean (SD) | N (%) or mean (SD) |
| Sex  Male  Female | 181,618 (58.90)  126,736 (41.10) | 147,085 (57.77)  107,504 (42.23) | 34,533 (64.23)  19,232 (35.77) |
| Mean age, SD  Age group (years old)  10-24  25-59  >60 | 30.68 (13.07)  110,378 (35.80)  190,709 (61.85)  7,267 (2.36) | 29.46 (12.32)  97,592 (38.33)  153,720 (60.38)  3,277 (1.29) | 36.52 (14.82)  12,786 (23.78)  36,989 (68.80)  3,990 (7.42) |
| Education Level (years of education)  Never studied  Pre-school  Primary school or less (<= 5 years)  Junior high school (6- 10 years)  High school (10-12 years)  College/university (>=13 years)  Missing data | 74,035 (24.01)  3,151 (1.02)  102,877 (33.36)  92,848 (30.11)  26,058 (8.45)  1,359 (0.44)  8,026 (2.60) | 58,374 (22.93)  2,440 (0.96)  86,024 (33.79)  80,181 (31.49)  19,170 (7.53)  778 (0.31)  7,622 (2.99) | 15,661 (29.13)  711 (1.32)  16,853 (31.35)  12,667 (23.56)  6,888 (12.81)  581 (1.08)  404 (0.75) |
| Race  White  Black  Asian  Mixed race/ Brown  Indigenous  Missing data | 129,373 (41.96)  25,805 (8.37)  905 (0.29)  122,840 (39.84)  690 (0.22)  28,741 (9.32) | 105,109 (41.29)  22,141 (8.70)  704 (0.28)  104,025 (40.86)  643 (0.25)  21,967 (8.63) | 24,264 (45.13)  3,664 (6.81)  201 (0.37)  18,815 (34.99)  201 (0.09)  6,774 (12.60) |
| Location of residence  Rural  Urban  Missing data | 49,638 (16.10)  247,876 (80.39)  10,840 (3.52) | 43,620 (17.13)  204,334 (80.26)  6,635 (2.61) | 6,018 (11.19)  43,542 (80.99)  4,205 (7.82) |
| Brazilian regions  Southeast  Northeast  Central-West  South  North  Missing data | 101,650 (32.97)  77,105 (25.01)  21,423 (6.95)  98,763 (32.03)  377 (2.93)  377 (0.12) | 82,493 (32.40)  67,309 (26.44)  16,795 (6.60)  79,989 (31.42)  7,683 (3.02)  320 (0.13) | 19,157 (35.63)  9,796 (18.22)  4,628 (8.61)  18,774 (34.92)  1,353 (2.52)  57 (0.11) |
| Household characteristics  Water supply  Public network (running water)  Well, natural sources, or other  Missing data  Waste  Public collection system  Burned, buried, outdoor disposal, or other  Missing data  Sanitation  Public network  Septic tank  Homemade septic tank  Ditch or other  Missing data  Construction materials  Bricks/cement  Wood, other plant materials, or other  Missing data | 233,635 (75.77)  61,319 (19.89)  13,400 (4.35)  243,476 (78.96)  51,478 (16.69)  13,400 (4.35)  151,939 (49.27)  45,656 (14.81)  64,384 (20.88)  31,585 (10.24)  14,790 (4.80)  217,627 (70.58)  77,339 (25.08)  13,388 (4.34) | 191,827 (75.35)  54,417 (21.37)  8,345 (3.28)  200,245 (78.65)  45,997 (18.07)  8,347 (3.28)  123,362 (48.46)  38,368 (15.07)  54,417 (21.37)  29,151 (11.45)  9,291 (3.65)  178,852 (70.25)  67,401 (26.47)  8,336 (3.27) | 41,808 (77.76)  6,902 (12.84)  5,055 (9.40)  43,231 (80.41)  5,481 (10.19)  5,053 (9.40)  28,577 (53.15)  7,288 (13.56)  9,967 (18.54)  2,434 (4.53)  5,499 (10.23)  38,775 (72.12)  9,938 (18.48)  5,052 (9.40) |
| Isolation  Lives with someone else  Lives alone | 302,868 (98.22)  5,486 (11.78) | 252,263 (99.09)  2,326 (0.91) | 50,605 (94.12)  3,160 (5.88) |
| Year of registration on CadÚnico  2001  2002  2003  2004  2005  2006  2007  2008  2009  2010  2011  2012  2013  2014  2015 | 1,635 (0.53)  73,607 (23.87)  81,213 (26.34)  30,226 (9.80)  14,851 (4.82)  33,482 (10.86)  17,306 (5.61)  11,268 (5.61)  9,438 (3.65)  9,344 (3.06)  7,463 (3.03)  10,115 (2.42)  4,712 (1.53)  2,915 (0.95)  779 (0.25) | 1,374 (0.54)  66,421 (26.09)  69,047 (27.12)  26,584 (10.44)  13,007 (5.11)  29,219 (11.48)  14,641 (5.75)  8,893 (3.49)  7,392 (2.90)  6,717 (2.64)  3,678 (1.44)  4,008 (1.57)  2,128 (0.84)  1,194 (0.47)  286 (0.11) | 261 (0.49)  7,186 (13.37)  12,166 (22.63)  3,642 (6.77)  1,844 (3.43)  4,263 (7.93)  2,665 (4.96)  2,375 (4.42)  2,046 (3.81)  2,627 (4.89)  3,785 (7.04)  6,107 (11.36)  2,584 (4.81)  1,721 (3.20)  493 (0.92) |
| Year of hospitalisation  2008  2009  2010  2011  2012  2013  2014  2015 | 41,168 (13.35)  36,265 (11.76)  37,640 (12.21)  39,023 (12.66)  39,799 (12.91)  39,350 (12.76)  38,555 (12.50)  36,554 (11.85) | 34,980 (13.74)  31,001 (12.18)  32,376 (12.72)  33,381 (13.11)  32,808 (12.89)  31,760 (12.48)  30,349 (11.92)  27,934 (10.97) | 6,188 (11.51)  5,264 (9.79)  5,264 (9.79)  5,642 (10.49)  6,991 (13.00)  7,590 (14.12)  8,206 (15.26)  8,620 (16.03) |
| Length of hospitalisation (days)  Less than 5 days  6 - 13 days  14 days and over | 75,408 (24.46)  86,296 (27.99)  146,650 (47.56) | 62,529 (24.56)  72,010 (28.28)  120,050 (47.15) | 12,879 (23.95)  14,286 (26.57)  26,600 (49.47) |

Abbreviations: BFP - Bolsa Família Programme.
