# Supplementary material for: Conditional cash transfers and mortality in people hospitalised with psychiatric disorders: A cohort study of the Brazilian Bolsa Família Programme
Source: PLoS Med. 2024 Dec 2;21(12):e1004486. doi: 10.1371/journal.pmed.1004486 (PMC11649113; doi:10.1371/journal.pmed.1004486)
Supplement: S9 Table — (DOCX) [file pmed.1004486.s019.docx]

**S9 Table. Description of year and length of hospitalisation overall and by Bolsa Família Programme participation, 2008-2015.**

|  | **Participants, No. (%)** | | |
| --- | --- | --- | --- |
| **Covariates** | **Overall**  **N= 69,901** | **BFP**  **N= 26,556 (37.99)** | **Non-BFP**  **N= 43,345 (62.01)** |
|  | N (%) or mean (SD) | N (%) or mean (SD) | N (%) or mean (SD) |
| Year of hospitalisation  2008  2009  2010  2011  2012  2013  2014  2015 | 18,328 (26.22)  13,679 (19.57)  11,848 (16.95)  10,554 (15.10)  7,278 (10.01)  4,686 (6.70)  2,719 (3.89)  809 (1.16) | 7,198 (27.10)  5,505 (20.73)  4,403 (16.58)  3,736 (14.07)  2,733 (10.29)  1,745 (6.57)  951 (3.58)  285 (1.07) | 11,130 (25.68)  8,174 (18.86)  7,445 (17.18)  6,818 (15.73)  4,545 (10.49)  2,941 (6.79)  1,768 (4.08)  524 (1.21) |
| Length of hospitalisation (days)  Less than 5 days  6 - 13 days  14 days and over | 16,062 (22.98)  18,685 (22.98)  35,154 (50.29) | 6,826 (25.70)  7,465 (28.11)  12,265 (46.19) | 9,236 (21.31)  11,220 (25.89)  22,889 (52.81) |

Abbreviations: BFP - Bolsa Família Programme
