# Supplementary material for: Conditional cash transfers and mortality in people hospitalised with psychiatric disorders: A cohort study of the Brazilian Bolsa Família Programme
Source: PLoS Med. 2024 Dec 2;21(12):e1004486. doi: 10.1371/journal.pmed.1004486 (PMC11649113; doi:10.1371/journal.pmed.1004486)
Supplement: Attachment — Submitted filename: Point by Point_PlosMedicine.docx [file pmed.1004486.s020.docx]

Dear Dr. Syba Sunny,

Thank you for giving us the opportunity to submit a revised draft of our manuscript. We appreciate the time and effort that you and the reviewers have dedicated to providing your valuable feedback on our work.

We were able to incorporate all the suggestions provided and we now have a more robust and readable manuscript.

Below is a point-by-point response to the editors' and reviewers' comments and requests.

Please note that the line numbers in this document refer to the "marked-up copy" version of the manuscript.

Cordially,

The authors

| **EDITORIAL COMMENTS** | **ANSWERS** |
| --- | --- |
| As you will see, the reviewers found your paper interesting. However, there were requests for further information and clarification of various points. In addition, the statistical reviewer (Reviewer 1) raised several important concerns about the study's design and analysis. Nevertheless, we remain interested in your work. After discussing the paper with the editorial team, I'm pleased to invite you to revise the paper in response to the reviewers' comments and our editorial requests (below). We plan to send the revised paper to some or all of the original reviewers*, and of course we cannot provide any guarantees at this stage regarding publication.  When you upload your revision, please include a point-by-point response that addresses all of the reviewer and editorial points, indicating the changes made in the manuscript and either an excerpt of the revised text or the location (e.g. page and line number) where each change can be found. Please submit a clean version of the paper as the main article file and a version with changes marked should as a marked-up manuscript. Please also check the guidelines for revised papers at http://journals.plos.org/plosmedicine/s/revising-your-manuscript for any that apply to your paper. | Done. |
| We thank you for submitting your valuable work to PLOS Medicine. We note your unique data set, but, equally, agree with all the points raised by the reviewers. As such, we require that you address all the reviewer and editorial comments in full, before we can consider your paper further.  1) Data Availability:  Please revise your data availability section. At present, there is missing information – please replace the parts with XXX with the relevant information. If the data are not freely available, please describe briefly the ethical, legal, or contractual restriction that prevents you from sharing it. Please also include an appropriate contact (web or email address) for inquiries (this cannot be a study author). Further information is available from http://journals.plos.org/plosmedicine/s/data-availability | We have included this information in the submission form:  The code book and analytical code will be made available upon request to the corresponding author via email: camila.bonfim@fiocruz.br. The data analyzed in this study is hosted by the Centre of Data and Knowledge Integration for Health (CIDACS). Full access to the data is restricted due to its sensitive nature and the exclusive licensing agreement for its use in this study. The privacy regulations set by the Brazilian Ethics Committee prohibit the public availability of this data. However, upon reasonable request, and provided that all ethical and legal requirements are met, the institutional data curation team can make the data available. Further information can be obtained at <https://cidacs.bahia.fiocruz.br/acesso-aos-dados/>. |
| 2) Reporting guidance: Please report your study according to the relevant guidance which can be found here https://www.equator-network.org/reporting-guidelines/ | Done. We included the strobe statement for observational studies in the supplement. |
| 3) Statistical reporting: (Note that not all of the following instructions will apply to your work, but please review all points and action as appropriate.) Please quantify the main results with 95% CIs and p values. When reporting p values please report as <0.001 and where higher as p=0.002, for example. When reporting 95% CIs please separate upper and lower bounds with commas instead of hyphens as the latter can be confused with reporting of negative values. Please include the actual amounts and/or absolute risk(s) of relevant outcomes (including NNT or NNH where appropriate), not just relative risks or correlation coefficients. (example for absolute risks: PMID: 28399126). Please include any important dependent variables that are adjusted for in the analyses. | Done. |
| 4) Prespecified analysis plan/study protocol: Did your study have a prospective protocol or analysis plan? Please state this (either way) early in the Methods section.  a) If a prospective analysis plan (from your funding proposal, IRB or other ethics committee submission, study protocol, or other planning document written before analyzing the data) was used in designing the study, please include the relevant prospectively written document with your revised manuscript as a Supporting Information file to be published alongside your study, and cite it in the Methods section. A legend for this file should be included at the end of your manuscript.  b) If no such document exists, please make sure that the Methods section transparently describes when analyses were planned, and when/why any data-driven changes to analyses took place.  c) In either case, changes in the analysis-- including those made in response to peer review comments-- should be identified as such in the Methods section of the paper, with rationale.  For all observational studies, in the manuscript text, please indicate: (1) the specific hypotheses you intended to test, (2) the analytical methods by which you planned to test them, (3) the analyses you actually performed, and (4) when reported analyses differ from those that were planned, transparent explanations for differences that affect the reliability of the study's results. If a reported analysis was performed based on an interesting but unanticipated pattern in the data, please be clear that the analysis was data-driven. | This study is part of a NIH funded project (mentioned in the paper), the protocol was previously published at (<https://pubmed.ncbi.nlm.nih.gov/36201469/> ). We have added the information regarding the protocol in the Methods section, and a specific protocol for these analyses is attached to the supporting document. |
| 5) Abstract layout: Please structure your abstract using the PLOS Medicine headings (Background, Methods and Findings, Conclusions), i.e. combine the 2 middle sections together to make a section called 'Methods and Findings'. | Done. |
| 6) Author summary: At this stage, we ask that you include a short, non-technical Author Summary of your research to make findings accessible to a wide audience that includes both scientists and non-scientists. The authors summary should consist of 2-3 succinct bullet points under each of the following headings: • Why Was This Study Done? Authors should reflect on what was known about the topic before the research was published and why the research was needed. • What Did the Researchers Do and Find? Authors should briefly describe the study design that was used and the study's major findings. Do include the headline numbers from the study, such as the sample size and key findings. • What Do These Findings Mean? Authors should reflect on the new knowledge generated by the research and the implications for practice, research, policy, or public health. Authors should also consider how the interpretation of the study's findings may be affected by the study limitations. In the final bullet point of 'What Do These Findings Mean?', please describe the main limitations of the study in non-technical language. The Author Summary should immediately follow the Abstract in your revised manuscript. This text is subject to editorial change and should be distinct from the scientific abstract. Please see our author guidelines for more information: <https://journals.plos.org/plosmedicine/s/revising-your-manuscript#loc-author-summary> | Done. It was included in the page 04 and 05. |
| 7) Introduction layout: Please address past research and explain the need for and potential importance of your study. Indicate whether your study is novel and how you determined that. If there has been a systematic review of the evidence related to your study (or you have conducted one), please refer to and reference that review and indicate whether it supports the need for your study. | Ok. |
| 8) Discussion layout: Please present and organize the Discussion as follows: a short, clear summary of the article's findings; what the study adds to existing research and where and why the results may differ from previous research; strengths and limitations of the study; implications and next steps for research, clinical practice, and/or public policy; one-paragraph conclusion. | Ok. |
| 9) Miscellaneous: Please revise your 'Short Title' – the word 'condicional' should be replaced with 'conditional'. | Done. |

| **Comments from the reviewers:** | **ANSWERS** |
| --- | --- |
| **Reviewer #1:** |  |
| Using data from the 100 Million Brazilian Cohort, this paper investigates the effect of conditional cash transfers on mortality among people previously hospitalised for any psychiatric disorder. A propensity score-based approach to analysis is taken. I have several questions about the analysis and other statistical aspects of the study. 1. This study is referred to as "quasi-experimental". However, I cannot determine what the experimental element of this study is. It would seem preferable to refer to this as an observational study. | Thank you for this valuable suggestion. We initially considered a quasi-experimental design for two main reasons: (1) the BFP has been recognized as a natural experiment originating from public policy^1^; (2) we used propensity scores to promote the comparability of treated and untreated groups, considering their measured pretreatment covariates and thereby controlling for confounding bias in estimating treatment effects. However, we acknowledge the study's observational nature since it was not specifically designed for this purpose. We have changed it to a cohort study to make it clearer. Adjustments have been made to the title (page 1, line 5), abstract (page 2, line 42), and method section (page 7, lines 203-204). |
| 2. Given that the patients included in the dataset for analysis may not have had a primary psychiatric disorder diagnosis, is it correct that all included patients were hospitalised due to a psychiatric disorder? It seems that some included patients were people hospitalised with a psychiatric disorder (rather than "for a psychiatric disorder"). | We very much appreciated your suggestion and changed it in the entire text. |
| 3. In the analysis presented in Tables 3 and 4, overall mortality is considered, but then mortality is divided up by cause. However, if an individual dies due an unnatural cause, they cannot die due to some other cause (for example). Thus, the results of the analysis presented in Table 3 for all apart from overall mortality do not have a useful interpretation. An appropriate analysis would account for competing risks of death by other causes, using a competing risks model. | Thank you for this excellent suggestion. We conducted the competing risks model and selected it as the final model. Accordingly, we have adapted the abstract, methods (page 15, lines 345-361), results (page 19, lines 448-455), and Tables 3 and 4 (now Tables 2 and 3, respectively) (pages 45, 46). All results found were similar to our previous models. We maintained Poisson models as a sensitivity analysis (Appendix 5.4, Table 11). |
| 4. It is unclear what the propensity score-based analysis that was applied here was - I could not access the Appendix, where additional details may have been supplied. What is clear is that a propensity score model for receipt of BFP was fit. However, the authors mention that IPTW was applied, but the details presented here do not align with a standard IPTW analysis. In such an analysis, all individuals would receive a weight of one over the probability that they received their actual "treatment" (here a cash transfer or not). Instead, here those participants who did receive the cash transfer had a weight of 1, and those participants who did not receive the cash transfer had another weight. I am not sure what is meant by "E(ps) is the probability if receiving the programme in the population": is this the average propensity score? The following approach is recommended: a. Assess the common support condition by comparing the range of propensity scores in each exposure group, and exclude participants outside the range of common support. b. Generate IPTWs following the formulas in reference 39. c. Calculate standardised differences in the weighted space (i.e. after applying the IPTWs). It is impossible to determine how well the propensity score model has performed without the calculation of these standardised differences. d. If standardised difference have reduced sufficiently, then the weighted outcome regression model can be it. This will include the exposure as the single covariate. Including the IPTWs as a covariate is not a recommended approach. | Thank you for your comment. We recognized the absence of this information and have improved it in the text. For the main analysis, we applied IPTW as a propensity-based method, as outlined in our protocol^2^, and a method applied in other studies using the 100 Million Brazilian Cohort^3 4 5 6^. We also conducted Kernel Weighting and Stabilized Inverse Propensity Scores (SIPTW) as a robustness analyses to evaluate if the results hold with other approaches for the same dataset and are not an artefact created with the IPTW weights. Regarding the weights estimated through IPTW, individuals who received the treatment (BFP beneficiaries) were given weights equal to the inverse of their propensity scores (1/PS). In contrast, those who did not receive the treatment were given weights equal to the inverse of one minus their propensity scores [PS/(1 – PS)] (reference). In the previous version manuscript, "E(ps)" represented the probability of receiving the treatment (the Bolsa Familia Programme). To clarify this, we have changed it to "PS".  We followed the recommended approach:  a. We have assessed the Common Support condition, and the graphs have been added to the Supplement (Appendix 4, Figure 03).  b. We generated IPTW following the formulas outlined in reference 39 mentioned in the paper^7^. After that, we identified extreme weights and corrected this problem using specified thresholds based on weight distribution for the 99th percentile according to the recommendation of the literature^8 9^. We have now added this information to the Methods Section (pages 14 and 15, lines 334-361).  c. We assessed the standardized differences before and after applying the IPTW. We have now added this information to the Methods section (page 13, lines 315-318) and Supplement (Appendix 4, Table 04)  d. Compared with baseline characteristics, the two groups became more similar after applying the IPTW approach (standardized differences of less than 0.1) (Appendix 4, Table 04). We clarify that the IPTW weight was not introduced in the final model as an adjusted covariate but applied as the weight function in Stata. We have now added this information to the Methods section (page 15, line 354).  After that, competitive risk models were estimated. This approach allows us to estimate the hazard ratio of the studied outcomes among beneficiaries (exposed groups) compared with non-beneficiaries (unexposed groups). We have now tried to clarify these steps in the Methods Section (pages 14 and 15) and the Supplement (Appendix 4). Finally, we confirm that we have not included IPTWs as ponderation in the models, following previous studies ^4 5 8 9^. |
| 5. I do not understand how the ATT was calculated, how the stabilised versions of the IPTWs were calculated or how the kernel matching approach was applied. More details are required. | We recognize the absence of this information and have added it to the text as follows:   1) We ran Stabilized Inverse Propensity Scores (SIPTW) approaches, which is a modification of the IPTW approach that incorporates stabilization of the weights to improve the numerical stability of the estimation process^8^; we estimated SIPTW weights for non-beneficiaries using the formula (1–Pt) / (1–Psmul) and for beneficiaries using the formula Pt /Psmul, where "Pt" is the marginal probability of treatment in the population and "Psmul" is the propensity score obtained from the multivariable logistic regression adjusted for covariates; we did the same truncation process in very extreme weights and ran multivariable competitive risk models using the SIPTW weights as a covariate in the final model as well as year and length of the hospitalisation; 2) Second, we estimated Kernel Matching (KM) approach, which establishes a weighting scheme for all untreated units, assigning greater weights to units closer to those treated units with which they will be matched^8^. This method was matched by weights estimated based on the propensity scores; pairs of beneficiaries and non-beneficiaries were matched by year of registration on the cohort, and the PS was matched with kernel weight for the same covariates. Then, we estimated the Average Treatment Effect on the Treated (ATT) (Appendix 5, Tables 05-08). Finally, the weight was introduced in the competitive risk models as a covariate as well as year and length of the hospitalisation. |
| 6. How many individuals were excluded due to missing data? | In the final model, we excluded 11,996 individuals due to missing information in any of the variables included in the models. It represents approximately 17% of the total population. To check the robustness of our results, we repeated the final model by treating missing data as a separate category, following the recommendation of Ali et al ^8^ and other studies using 100 million Brazilian cohort^3 4 5 6^. The results remained similar and have been included in the supplement (Appendix 5.3, table 10). |
| 7. How were the standardised mean differences calculated for the categorical characteristics? Generally an SMD would be calculated for each level of the characteristic. | We appreciate the opportunity to clarify this point. A difference between the groups before applying the PS was observed, reinforcing the importance of using PS to make the groups comparable (Table 1). To assess the balance between the treated and non-treated groups, we examine the differences in the proportions of the variables before and after IPTW weighting. We highlighted that we assessed the balance for categorical covariates by looking at the difference in the proportion of individuals at each level of the covariates between treated and untreated groups^8^. We assessed continuous covariates by examining the difference in the mean covariate values between treated and untreated groups^8^. We also ran “Covbal” command at Stata to evaluate each covariates specified, and assess balance between treated and untreated groups, and means using standardized difference. This analysis was added in the Supplement (Appendix 4, Table 04). |
| 8. Given my comments on the analysis above, it is difficult to determine whether the conclusions drawn in this study are supported by the data. | Your concerns are valid, and to address them, we have run all the analyses you suggested, including competitive risk analysis, and all the results remained similar. Furthermore, we added a more detailed explanation of the calculation of propensity scores and ITPW, as well as the sensitivity analysis. |

| **Reviewer #2:** | **ANSWERS** |
| --- | --- |
| This is an interesting article using a quasi-experimental study design to examine whether enrollment in a conditional cash transfer program was associated with reduced mortality among Brazilians previously hospitalized for a psychiatric illness. I am generally in agreement with the analysis and its interpretation. However, I did have the following questions: |  |
| 1) Why was the sample restricted to those with a psychiatric hospitalization only once during the study period? Researchers provide a rationale but I'm unsure how does this differ from other studies that looked at the overall Brazilian Bolsa Família Programme enrollment and mortality risk from different causes. | We appreciate this observation and clarify that we focused on a specific population with heightened vulnerability due to mental disorders. Other studies on the BFP have already explored its effects on the general population's mortality. We expected a higher risk of death in our study compared to other studies from the 100 million Cohort because our population was more vulnerable, being both impoverished and mentally ill. We chose to include only individuals with a single hospitalisation to ensure sample comparability, as multiple hospitalisations could indicate greater severity of mental disorders. By doing so, we excluded 342,992 cases, which is about 45% of our population. Finally, the period of the study was limited to the data availability. |
| 2) Did you stratify according to whether the participant primary vs. secondary diagnosis was for psychiatric disease to see if results differ according to disease severity? | We appreciate this question, but unfortunately, we do not have a sufficient sample size to perform this analysis, as only 5.07% of the sample was hospitalised with a psychiatric disorder as a secondary diagnosis. We also clarify that we do not have information on the severity of the diagnosis, only whether the individual was hospitalised with this diagnosis. |
| 3) Your propensity score did not control for any health-related covariates? Is health a strong predictor for enrollment in this CCT program? | Thank you for the opportunity to clarify this point. The enrollment in the BFP is based on the family's per capita income, categorized into extreme poverty and poverty. Therefore, whether a family receives the benefit depends on their poverty status. To assess the likelihood of receiving BFP, we utilized a set of available family socioeconomic variables from our dataset to characterize their poverty status. While we acknowledge the importance of health variables, socioeconomic factors appear to be the most relevant for determining treatment assignment for BFP, as poverty status is the primary criterion for programme eligibility. Because of this, we chose sociodemographic and socioeconomic covariates to construct the propensity score. |
| 4) Participants in your study only applied for the CCT program after their psychiatric hospitalization - would the initial hospitalization influence enrollment in CCT in any way? | We selected only those who applied to the programme after hospitalisation to reduce potential biases related to better socioeconomic conditions that could improve mental health and reduce hospitalisation rates ^10^. The requirement to participate in the programme is exclusively financial (families who receive a household income less than USD25 or 100 Brazilian reais per capita). Therefore, we shouldn't expect psychiatric hospitalisation to increase the chances of participation unless health services start referring patients for financial support. It is precisely what our results suggest in terms of policy impact. However, automatic referral in psychiatric units is still not implemented in Brazil. |
| 5) Minor - I am confused when the researchers write about hospitalized beneficiaries in the text because I thought all the participants were hospitalized. | Thank you for this observation. You are right; all the participants were hospitalised. We used the term "hospitalised beneficiaries" to emphasize the difference between the population of this study and other studies using the 100 million Cohort, which includes the general population. However, we have now revised the paper to exclude "hospitalised". |
| 6) Minor - What is the rationale for the age groupings? | Thank you for the opportunity to clarify this point. The categorization of age in our study aligns with our previously published research protocol^2^ and other studies^3 11 12^. |

| **Reviewer #3:** | **ANSWERS** |
| --- | --- |
| This manuscript reports findings from an interesting analysis on a large Brazilian database in which the authors investigated mortality after a psychiatric hospitalisation and investigated associations with conditional cash transfer payments. My comments are as follows: |  |
| 1. Line 121 - for a journal of this general nature, I think a sentence could usefully be added on what a CCTP entails, beyond simply saying that it's a social policy; in particular, some description is needed as to whether it's a tightly defined intervention internationally or is describing something much broader and heterogeneous. Currently the paper assumes that this will be familiar territory to all readers. | We recognized the absence of this information and have improved it on page 07.  We included the following: Cash transfer programmes are social policies commonly used in LMICs to alleviate extreme poverty and poverty-related outcomes such as food insecurity. These programmes can present considerable heterogeneity among countries related to transfer value, frequency and duration of the benefit, presence of conditionalities such as health and schooling attendance, targeting and eligibility criteria as well as implementation systems (government or private)^13 14^. |
| 2. Lines 146-157 - likewise, there are quite a lot of assumptions about readers knowledge of Brazilian healthcare. For interpretation, it would probably be important to be clear what healthcare options are available in Brazil and what proportion of people will register for social assistance (and thus, presumably, appear on the source dataset). | We recognized the absence of this information and included it on page 09, as follows:  Brazil presents one of the world's largest and most complex public health systems, ranging from primary to tertiary levels, the last one where hospital care is provided. Their access is comprehensive, universal, and free for the country's entire population. Although a private supplementary system exists, over 70% of the Brazilian population relies on the public health system, particularly the poorest segments of society^15^. |
| 3. Figure 1 - the substantial drop from n=152,862 to n=71,068 is a little concerning. Am I correct in assuming that 'registration date after follow-up has ended' represented an indication of data errors? If so, this seems quite a high level and it would be helpful if there was some assurance of data quality for the remainder. | Thank you for the opportunity to clarify this point. We apologize for any confusion regarding the study population flowchart. The 100 million Cohort includes data from 2001 to 2018, but the other databases (SIH and SIM) lack complete information for this entire period. For our study, we have data from all three sources only between 2008 and 2015. Therefore, we improved Figure 1 and the participants' section (page 10) to clarify the exclusion of individuals outside the study period. We also added more details to the flowchart in the supplement (Appendix 2). |
| 4. Is BFP registration at individual or household level? If at household level, was any procedure deployed to account for clustering? | Thank you for this valuable observation and the opportunity to clarify it. The BFP is a family benefit, so the information is replicated for each family's individual. We calculated the ICC to identify any potential cluster effects; however, the correlation was founded to be low^16^. (ICC = 0.016, 95% CI = 0.001, 0.620). Therefore, we did not control for the cluster effects. We have now included this analysis in the supplement (Appendix 6, Table 12). |
| 5. Table 2 - is there any particular reason for the inconsistency in the ordering of columns between the two comparison groups? | We apologize for the confusion in the ordering of columns in Table 2. We have now corrected it and move it for the supplement following recommendation of the Reviewer 4. |
| 6. Is there any information that can be provided on the extent of propensity score overlap between the two comparison groups? Is it appropriate to be including people with 0% or 100% propensity scores (assuming there were some of them)? Or do the sensitivity analyses cover this scenario? | Thank you for the question. We did not have propensity scores of 0 or 1. Among beneficiaries, the lowest value was 0.101, and the highest was 0.867, while among non-beneficiaries, the lowest value was 0.059, and the highest was 0.767. We have added a summary of the PS and the common support graph in the supplement for better visualization (Appendix 4, Table 03). |
| 7. As a general comment, for a paper whose analyses are focused on public health and policy, it would be worth considering the inclusion of metrics with stronger communication in that field. From the results available, wouldn't it be possible to include a PAF equivalent (i.e., the proportion of deaths that could theoretically have been prevented with the intervention, assuming causality) and/or a NNT equivalent (number receiving the intervention required to result in one fewer deaths over a given follow-up period)? | Thank you for this excellent suggestion. We calculated the PAF. We used the formula PAF= [P(HR-1)]/[P(HR-1)+1]^17^ where P was the incidence of the BFP (exposition) in the population and HR the hazard risk estimated for the association between BFP and overall mortality. Following a previous publication of our group^18^, we estimated unadjusted and adjusted PAF using the punafcc package in Stata, which uses Cox Regression and a 95% Confidence Interval. For the adjusted model, we included the same covariates as the final model. In the unadjusted analysis, we found that the BFP prevented 18% (95% CI 16%, 20%) of deaths among individuals hospitalised with psychiatric disorders, while in the adjusted analysis, it reduced to 4% (95% CI 0,6%, 7,1%). We added the formula used for the calculation in the methods section (pages 15, 16) and the findings in the results section (page 19), abstract (page 3) and author summary (page 4). |

| **Reviewer #4:** | **ANSWERS** |
| --- | --- |
| This manuscript is based in the 100 Million Brazilian Cohort, a dynamic cohort representing people who registered for CadUnico, a system used to apply for social assistance in Brazil. The authors examined whether a conditional cash transfer benefit, the Bolsa Familia Program (BFP) reduced mortality after a single hospitalization for a primary or secondary psychiatric diagnosis during 2008-2015. Importantly, the conditions for receiving the BFP cash transfer include participation education, health and other social programs.  The study sample comprised those who registered for CadUnico and applied for BFP after their psychiatric hospitalization and before 2015. Within the study sample, they compared mortality rates up to 2015 for those who received vs did not receive BFP. Those who received BFP were followed from the time they received it, and those who did not receive BFP were followed from the time they registered for CadUnico. BFP was associated with a substantially reduced mortality rate overall. This was primarily driven by a reduced mortality for "natural" causes, although there was also a trend for "unnatural" causes such as suicide.  These are the first results to suggest that a broad program of social assistance, not directed at psychiatric patients in particular, has major benefits in reducing mortality among psychiatric patients after discharge. It has broader significance too, opening the question of whether such assistance could reduce the well established high mortality rate among all people with psychiatric disorders.  Overall, the study appears to be solid, once one understands what the authors did. The presentation, however, is somewhat confusing. It took us quite some time to understand what they did. For most readers, who will not be familiar with the 100 million cohort nor the BFP, it would be much more difficult. In that regard we have several comments: 3 main ones and then several smaller ones. We have not included the many ways in which the study could be expanded in scope or in detail, because we recognize that the paper is already reporting an important result, and further results could be in future papers. |  |
| Main Comments  First The authors need to clarify the study sample in the text and in Figure 1. The large number who were excluded due to registering for CadUnico after 2015 is not noted in the text under Participants where Figure 1 is first referred to. In Results, where the authors again refer to Figure 1, they only note that they excluded persons who did not meet inclusion criteria, without noting that the largest number were excluded due to registration after 2015 (this can be found in the Figure 1 but not easily). It is not clear why these persons were considered participants in the first place, but whatever they call them, what is important is to describe the study sample upfront and highlight the key points clearly, such as this one. | Thank you for the opportunity to clarify this point. We apologize for any confusion regarding the study population flowchart. The 100 million Cohort includes data from 2001 to 2018, but the other databases (SIH and SIM) lack complete information for this entire period. For our study, we have data from all sources only between 2008 and 2015. Therefore, we improved the figure 1 and participants section (page 10, 11) to clarify the exclusion of individuals outside the study period. We added more detail to the flowchart in the supplement (Appendix 2). |
| Note in the manuscript that the profile of individuals hospitalized for psychiatric reasons in other studies differs markedly from the cohort studied here (see Rocha et al, 2021, Revista de saude publica). The authors should also note how that might be due to the selection criteria for this study. | Thank you for your insightful comment. We appreciate your observation regarding the differences between the profile of individuals hospitalised with psychiatric reasons in our study compared to those in other studies, such as Rocha et al. (2021)  Our manuscript's selection criteria focused exclusively on individuals who registered at CadUnico after hospitalisation. This criterion was intentionally chosen to reduce potential biases related to better socioeconomic conditions that could improve mental health and reduce hospitalisation rates^10^. Consequently, this may have resulted in a cohort that differs from those used in other studies with different selection criteria.  We have now highlighted this distinction in our manuscript, discussing how our specific selection criteria might contribute to the observed differences in the profile of hospitalised individuals compared to Rocha et al study (Discussion section, page 25). |
| Other points that need to be clarified about the study sample include: were these "first ever" psychiatric hospitalizations, or "first during 2008-2015"? | Thank you for the opportunity to clarify this. The study sample included the first hospitalisations between 2008 and 2015 when complete data was available. We did not have information on whether this admission was the first ever. We have updated the text with this information in the Participants section (page 10). |
| what were the main reasons why some who applied for BFP received it or did not receive it? | Thank you for this question. We clarify that the eligibility criteria for receiving the BFP are a monthly household income of less than BRL 70.00 (USD 17.00) or BRL 140.00 (USD 34.00) for households with a child, adolescent or pregnant woman. This information is on Method section (page 12). |
| for those who received BFP, how long was the period between hospitalization and receipt? | Thank you for the question. The average time of receiving BFP after hospitalisation was 2.86 years (SD=1.85). We have added this information in the Results section (page 18). |
| what proportion were in psychiatric versus general hospitals? were there differences in mortality between these two groups? between those with primary vs secondary psychiatric diagnoses? | Unfortunately, we do not have information about whether the hospital is general or psychiatric. Regarding the diagnosis, only 5.07% were admitted with a secondary diagnosis of a psychiatric disorder. We have added this information to the Methods (page 10). |
| When the answers to these questions are not available, that should simply be stated. | Thank you, we have now stated them in the manuscript. |
| Second  In the introduction or Discussion, it would be helpful to further situate the reader in terms of the unified healthcare system in Brazil, who it serves, and the general psychosocial attention policies (e.g., Law 10.216/2001, implementation of CAPS, reduction of hospital beds in psychiatric hospitals and increase in beds in general hospitals). | Thank you for this valuable observation. We included this information in the Discussion (pages 21 and 22):  “In Brazil, the public healthcare system for individuals with psychiatric disorders is provided through the Psychosocial Care Network, which includes services such as primary care, community-based mental health centers (called psychosocial care centers, or CAPS, in Brazil), emergency and urgent care network, residential services, and hospitals^19^. The Brazilian mental health reform has increasingly prioritized mental health care in the CAPS, progressively reducing psychiatric hospital beds, which were the primary option before the reform.”^20^ |
| Also, clarify how individuals who are registered in the CadUnico differ from the entire population even though they comprise over 60% of Brazil's population. One sentence referencing the original 100 million cohort paper should suffice to situate the reader. | Thank you for this valuable observation. We included this information in the Discussion (page 25):  “Compared to the Brazilian population, this cohort over-represents young people and women due to the BFP target population”.  With regard to the cohort, we have in the Methods (page 9) the following statement: "The 100 Million Brazilian Cohort is a dynamic cohort, comprised of individuals registered on CadÚnico, which is the primary system used for anyone applying for social assistance in Brazil."^21^ |
| Line 364: this may not be clear to international readers. A potential alternative "community based mental health centers (called psychosocial care centers, or CAPS, in Brazil)" | We appreciate this suggestion and have now changed the text accordingly (Discussion section, page 21, line 505). |
| Space could be made by implementing the third suggestion below.  Third The main results are in Tables 3 and 4, and Table 2 is not needed for the main text. Since Table 2 has only unadjusted results, it should be made a supplement. Table 2 provides information that might be needed to fully evaluate the study. Anything that needs to be highlighted- such as differences between BFP and non-BFP in person-years at risk- could be briefly alluded to in the text. However, presenting the unadjusted results in detail in the main text and table 2, before the main results, is quite confusing for readers. | We appreciate the suggestion and have moved Table 2 to the supplement (Appendix 3, Table 01). |
| Since groups differ on person-years at risk, could the authors explain the choice of IRRs rather than doing a survival analysis such as proportional hazards? We don't insist on doing a survival analysis, but think some rationale for the approach chosen is needed. | Thank you for this excellent suggestion. We conducted a survival analysis using proportional hazard and competitive risk models and adapted tables and results accordingly. |
| Smaller points:  Line 84: we think mortality risk should be mortality rate. | We agree with the change and have updated the text (Introduction section, page 6, line 133). |
| Line 112: note lack of LMIC data on mortality for psychiatric disorders. | We agree with the change and have updated the text (Introduction section, page 07, line 162). |
| Line 113: "approximately 62·2% of the global burden of suicide deaths may be attributed to psychiatric disorders". This statement derives from a paper but might be problematic because "attributing" suicide deaths to a single factor across the globe is fraught with problems. It seems unnecessary here though we leave this to the authors' discretion. | We agree with this observation, changed the verb "attributed" to "associated", and have updated the text (Introduction section, page 7, line 161). |
| Lines 114-116: include the role of psychiatric treatments in mortality (e.g., long term neuroleptic use) | We agree with this observation and have updated the text (Introduction section, page 7, line 164). |
| Line 131: note that the post-discharge period is particularly vulnerable, and that psychiatric hospitalizations themselves may increase all-cause mortality, as shown in many articles. | We agree with this observation and have updated the text (Introduction section, page 8, lines 186-188). We included:  “The contribution of CCTPs in this population would be particularly relevant, considering that there is an increased risk of death after discharge.”^22^ |
| Line 237 delete statement that SMDS >.1 indicated confounding. This would not be the correct approach, and indeed, they do not follow it in constructing the propensity scores. | We apologize for the confusion in writing and have improved the text to clarify how we used SMD. We clarified that we used SMD to identify imbalances in the distribution of covariates between treated and untreated groups. Values above 0.1 indicated this imbalance, prompting us to use propensity scores and IPTW approaches. We updated the text accordingly (Method section, page 13, lines 315-318). |
| Line 260: how was length of hospitalization obtained? Authors may be aware that the typical way to obtain that information is not reliable because administrators often circumvent the need to maintain the same AIH number for hospitalizations longer than 15 days and often generate a new number, which may cause problems calculating length of stay. Other authors have found ways to address this, please clarify how you dealt with this issue or if LOS was calculated in a different way | We appreciated the opportunity to explain this covariate better. It was calculated as follows: 1) Using the user identification number in the 100 million Brazilian cohort, we identified who had multiple hospital admission records (AIH); 2) We excluded all individuals with multiple records and retained only the record of the first hospital entry between 2008 and 2015; 3) We calculated the difference between the discharge date and the admission date of this first hospitalisation; 4) We computed this difference in terms of days and categorized it based on tertiles. We have added this explanation to the text (Method section, page 13, lines 297, 298). |
| Line 281: please provide rationale for excluding everyone who received Bolsa Familia before their hospitalization, as it seems that this would be an important group to examine- but might be beyond the scope of the study. | Thank you for the opportunity to clarify it. We selected only those registered at CadUnico after hospitalisation to reduce potential biases related to better socioeconomic conditions that could improve mental health and reduce hospitalisation rates^10^. We have now clarified it in the text (Method section, page 11, lines 254-257). |
| Line 305: interesting finding, could there be group differences because women were more likely to have been excluded from this cohort for being on benefits before hospitalization (e.g., because of pregnancy)? | We appreciate this valuable reflection. We analyzed the excluded sample (those registered before hospitalisation, N=308,354) and identified a similar profile. We compared the treated and untreated groups and observed the same proportion of these results. We have now added a descriptive table of this population in the supplement (Appendix 7, Table 13). We have also clarified in the manuscript that the reason for excluding people who were already registered (and therefore could receive or not receive the BFP) was to avoid potential selection bias. This exclusion helps to focus specifically on assessing the impact of the BFP after hospital discharge, considering the potential influence of BFP receipt on improving socioeconomic conditions and potentially reducing hospitalisation rates, as suggested in the literature^10^. |
| Line 331: change "there were effects" to "results were consistent with an effect" | We agree with this observation and have updated the text (Discussion section, page 20, lines 468, 469). |
| Line 365: consider explaining that a national policy gradually reduced the numbers of hospital beds in psychiatric hospitals and implemented community based care nationally starting in 2021. | We appreciated this observation and updated the discussion section (Discussion section, page 21, lines 503-509):  “In Brazil, the public healthcare system for individuals with psychiatric disorders is provided through the Psychosocial Care Network, which includes services such as primary care, community-based mental health centers (called psychosocial care centers, or CAPS, in Brazil), emergency and urgent care network, residential services, and hospitals^19^. The Brazilian health reform has increasingly prioritized mental health care in the CAPS, progressively reducing psychiatric hospital beds, which were the primary option before the reform.”^20^ |
| Lines 367-369: the national policy encourages hospitalizations in general hospitals and discourages psychiatric beds in psychiatric hospitals. Authors may be referring to the fact that smaller cities may not have professionals with the right expertise (psychologists, psychiatrists, occupational therapists, psychiatric nurses), or the appropriate space in general hospitals to serve the complex needs of this population | We appreciated this observation and updated the discussion section (Discussion section, page 22, lines 514-518):  "Although the provision of mental health care in general hospitals can bring benefits such as stigma reduction, increased service access, improved physical health care, and the possibility of multidisciplinary team care, the number of psychiatric beds available in these hospitals remains limited." ^20^ |
| Lines 455-456: national policy encourages general hospital admission vs psychiatric hospital, please revise. | We appreciated this observation and updated the discussion section (Discussion section, page 21, 22, lines 507-509):  "The Brazilian health reform has increasingly prioritized mental health care in the CAPS, progressively reducing psychiatric hospital beds, which were the primary option before the reform."^20^ |

Ezra Susser and Ana Florence


Any attachments provided with reviews can be seen via the following link:
********

-----------------------------------------------------------
COMMENTS FROM THE ACADEMIC EDITOR:

The Academic Editor was supportive of your work from the outset. She commented that she believed that you address a very important topic and also commented positively regarding your unique data set. She noted the concerns raised by the statistical reviewer, and encourages you to address these.

-----------------------------------------------------------
GENERAL EDITORIAL REQUESTS:

1. Please upload any figures associated with your paper as individual TIF or EPS files with 300dpi resolution at resubmission; please read our figure guidelines for more information on our requirements: http://journals.plos.org/plosmedicine/s/figures. While revising your submission, please upload your figure files to the PACE digital diagnostic tool, https://pacev2.apexcovantage.com/. PACE helps ensure that figures meet PLOS requirements. To use PACE, you must first register as a user. Then, login and navigate to the UPLOAD tab, where you will find detailed instructions on how to use the tool. If you encounter any issues or have any questions when using PACE, please email us at PLOSMedicine@plos.org.

2. Please ensure that the paper adheres to the PLOS Data Availability Policy (see http://journals.plos.org/plosmedicine/s/data-availability), which requires that all data underlying the study's findings be provided in a repository or as Supporting Information. For data residing with a third party, authors are required to provide instructions with contact information for obtaining the data. PLOS journals do not allow statements supported by "data not shown" or "unpublished results." For such statements, authors must provide supporting data or cite public sources that include it.

3. We ask every co-author listed on the manuscript to fill in a contributing author statement, making sure to declare all competing interests. If any of the co-authors have not filled in the statement, we will remind them to do so when the paper is revised. If all statements are not completed in a timely fashion this could hold up the re-review process. If new competing interests are declared later in the revision process, this may also hold up the submission. Should there be a problem getting one of your co-authors to fill in a statement we will be in contact. YOU MUST NOT ADD OR REMOVE AUTHORS UNLESS YOU HAVE ALERTED THE EDITOR HANDLING THE MANUSCRIPT TO THE CHANGE AND THEY SPECIFICALLY HAVE AGREED TO IT. You can see our competing interests policy here: http://journals.plos.org/plosmedicine/s/competing-interests.

-----------------------------------------------------------

To submit your revised manuscript please use the following link:

https://www.editorialmanager.com/pmedicine/

Your article can be found in the "Submissions Needing Revision" folder.

__________________________________________________
In compliance with data protection regulations, you may request that we remove your personal registration details at any time. (Use the following URL: https://www.editorialmanager.com/pmedicine/login.asp?a=r). Please contact the publication office if you have any questions.

**References**

1 Barreto ML. Invited commentary on quasi-experiments: Going beyond the observational and experimental dichotomy in epidemiological study design. *Curr Epidemiol Rep*. 2016; 3:259–61.

2 Machado DB, Azevedo JPA, Alves FJO, Castro-de-Araujo LFS, Silva ER, Fialho EMX, et al. The impact of social drivers, conditional cash transfers and their mechanisms on the mental health of the young; an integrated retrospective and forecasting approach using the 100 million Brazilian Cohort: A study protocol. *PLoS One*.2022; 17(10): e0272481.

3 Machado DB, Williamson E, Pescarini JM, Alves FJO, Castro-de-Araujo LFS, Ichihara MY, et al. Relationship between the Bolsa Família national cash transfer programme and suicide incidence in Brazil: A quasi-experimental study. *PLoS Med*. 2022; 19(5): e1004000.

4 Alves FJO, Ramos D, Paixão ES, Falcão IR, de Cássia Ribeiro-Silva R, Fiaccone R, et al. Association of Conditional Cash Transfers With Maternal Mortality Using the 100 Million Brazilian Cohort. *JAMA Netw Open*. 2023; 6(2):e230070.

5 Jesus GS, Pescarini JM, Silva AF, Torrens A, Carvalho WM, Junior EPP et al. The effect of primary health care on tuberculosis in a nationwide cohort of 7·3 million Brazilian people: a quasi-experimental study. *Lancet Glob Health*. 2022; 10(3): e390-e397

6 Ramos D, da Silva NB, Ichihara MY, Fiaccone RL, Almeida D, Sena S et al. Conditional cash transfer program and child mortality: A cross-sectional analysis nested within the 100 Million Brazilian Cohort. *PLoS Med*. 2021; 18(9): e1003509.

7 Williamson E, Morley R, Lucas A, Carpenter J. Propensity scores: from naive enthusiasm to intuitive understanding. *Stat Methods Med Res*.2012; 21(3): 273-293.

8 Ali MS, Prieto-Alhambra D, Lopes LC, Ramos D, Bispo N, Ichihara MY, et al. Propensity Score Methods in Health Technology Assessment: Principles, Extended Applications, and Recent Advances. *Front Pharmacol*. 2019; 10(973): 1-19.

9 Jesus GS, Pescarini JM, Silva AF, Torrens A, Carvalho WM, Junior EPP et al. The effect of primary health care on tuberculosis in a nationwide cohort of 7·.3 million Brazilian people: a quasi-experimental study. *Lancet Glob Health*. 2022; 10(3): e390-e397.

10 Pescarini JM, Campbell D, Amorim LD, Falcão IR, Ferreira AJF, Allik M, et al. Impact of Brazil's Bolsa Família Programme on cardiovascular and all-cause mortality: a natural experiment study using the 100 Million Brazilian Cohort. *Int J Epidemiol*. 2022 Dec; 51(6): 1847-1861.

11 Sawyer SM, Azzopardi PS, Wickremarathne D, Patton GC. The age of adolescence. Lancet Child Adolesc Health. 2018 Mar;2(3):223-228.

12 Brasil*. Estatuto do Idoso.* Brasília: Senado Federal; 2019.

13 Prencipe L, Houweling TAJ, van Lenthe FJ, Palermo T. Do Conditional Cash Transfers Improve Mental Health? Evidence From Tanzania's Governmental Social Protection Program. *J Adolesc Health*. 2021 Nov; 69(5):797-805

14 Evans-Lacko S, Araya R, Bauer A, Garman E, Álvarez-Iglesias A, McDaid D, et al. Potential mechanisms by which cash transfer programmes could improve the mental health and life chances of young people: A conceptual framework and lines of enquiry for research and policy. *Glob Ment Health (Camb).* 2023 Feb 21;10: e13.

15 Instituto Brasileiro de Geografia e Estatística (IBGE). *Pesquisa nacional de saúde*: 2019: informações sobre domicílios, acesso e utilização dos serviços de saúde: Brasil, grandes regiões e unidades da federação. Rio de Janeiro: IBGE; 2020.

16 Karry SM, Bland AM. The intraclass correlation coefficient in cluster randomisation. *BMJ*. 1998; 316: 1455.

17 Askari M, Namayandeh SM. The Difference between the Population Attributable Risk (PAR) and the Potential Impact Fraction (PIF). *Iran J Public Health*. 2020; 49(10): 2018-2019.

18 Rebouças P, Paixão ES, Ramos DO, Pescarini J, Pereira Junior E, Falcão IR et al. Ethno-racial inequalities on adverse birth and neonatal outcomes: a nationwide, retrospective cohort study of 21 million Brazilian newborns. *Lancet Regional Americas*. 2024; 37: 100833

19 Brasil. Ministério da Saúde. Portaria n. 3088 de 23 de dezembro de 2011. Institui a Rede de Atenção Psicossocial para pessoas com sofrimento ou transtorno mental e com necessidades decorrentes do uso de crack, álcool e outras drogas, no âmbito do Sistema Único de Saúde (SUS). Brasília: Ministério da Saúde; 2011.

20 Rocha HA da, Reis IA, Santos MA da C, Melo APS, Cherchiglia ML. Psychiatric hospitalizations by the Unified Health System in Brazil between 2000 and 2014*. Rev Saúde Pública*. 2021; 55:14.

21 Barreto ML, Ichiara MY, Pescarini JM, Ali MS, Borges GL, Fiaccone RL, et al. Cohort Profile: The 100 Million Brazilian Cohort. *Int J Epidemiol*. 2022; 51(2): e27-e38.

22 Swaraj S, Wang M, Chung D, Curtis J, Firth J, Ramanuj PP, et al. Meta-analysis of natural, unnatural and cause-specific mortality rates following discharge from in-patient psychiatric facilities. *Acta Psychiatr Scand*. 2019 Sep;140(3):244-264.
