# Supplementary material for: Conditional cash transfers and mortality in people hospitalised with psychiatric disorders: A cohort study of the Brazilian Bolsa Família Programme
Source: PLoS Med. 2024 Dec 2;21(12):e1004486. doi: 10.1371/journal.pmed.1004486 (PMC11649113; doi:10.1371/journal.pmed.1004486)
Supplement: Attachment — Submitted filename: Point by Point_PlosMedicine_2_v3.docx [file pmed.1004486.s021.docx]

Dear Dr. Syba Sunny,

Thank you for giving us the opportunity to resubmit a revised version of our manuscript. We greatly appreciate the time and effort that you and the reviewers have put into providing valuable feedback on our work.

We have incorporated all the suggestions provided and present a manuscript free of inconsistencies, as well as made a few insertions and English corretions to clarify some information.

Below is a point-by-point response to the editors' and reviewers' comments and requests.

Please note that the line numbers in this document refer to the "marked-up copy" version of the manuscript.

Cordially,

The authors

| **EDITORIAL COMMENTS** | **ANSWERS** |
| --- | --- |
| Comments from the academic editor:  The academic editor continued to be supportive of your manuscript and hopes that you can address the statistician's points. | Thank you. We appreciate it very much and have addressed all the points raised by the statistician. |

| **Comments from the reviewers:**  **# REVIEWER 1 (Statistician)** | **ANSWERS** |
| --- | --- |
| I thank the authors for their responses to my comments on the previous version of this manuscript. |  |
| However, I have noticed some inconsistencies throughout the manuscript and supplementary materials that require attention. Please note that line numbers in my comments refer to those on the tracked changes version of the manuscript. | Your observations were highly pertinent, and we have made efforts to include the requested information and align the data in the manuscript with that in the Appendix. |
| 1. Abstract: please change "competitive risk" to "competing risks" (here and on line 356 and Tables 2, 3, and in the Supplementary material) | Done. |
| 2. Line 315: IPTW estimation is mentioned before it is defined. Please define this acronym when first used. | Done. |
| 3. Is the primary aim to estimate the ATT (average effect in the treated), as stated on line 347? This should be stated early on in the Statistical Analyses section. If this is the case the authors were correct in their previous choice of weights: 1 for those individuals who did receive a cash transfer, and PS/(1-PS) for those who did not. However, I am confused since on line 389 it is stated that estimation of the ATT is done as sensitivity analysis (or is this just referring to one particular method for estimation of the ATT)?  I also note that the text in the manuscript regarding the weights should match that in the Appendix. | Thank you for your observation. The ATT is the primary aim of our analysis. We mentioned ATT estimation in line 389 because we also calculated it in the sensitivity analysis (Kernel Matching approach). This data is available in Appendix (S8, tables 5-8). We have now clarified this procedure in lines 381-385:  "Beneficiaries and non-beneficiaries were matched by year of registration in the cohort, and the propensity scores matched with kernel weights for the same covariates used in the final model generating the ATT (Appendix S5 Tables 5-8)."  Thank you for pointing this out, we have now corrected the values and mentioned in the manuscript that further information regarding the weight calculations is in the Appendix S4 Text. |
| 4. Are the SMDs in Table 1 for weighted or unweighted data? Please provide SMDs for the unweighted and weighted data in Table 1. Why do the SMDs in Table 1 not match those in the before IPTW column in S4 Table 4? | We appreciate the opportunity to clarify this point. The SMD, which was in Table 1 reflects only unweighted data. The values differ from those in S4 Table 4 because they are not the same analysis. In the Table 1, we calculated SMD using "sttdiff" command in Stata. However, this command does not allow us to calculate the SMD applying the weights. For this reason, we used the 'covbal' command to estimate the differences in proportions for each category between BFP beneficiaries and non-beneficiaries. Then, we can assess the balance between the treated and non-treated groups, examining the differences in the proportions of the covariates before and after IPTW weighting. We have now updated Table 1 using the data which were in S4 Table 4 and removed the SMDs to the table 1. Furthermore, we moved the data on the study population from Table 1 to Appendix S8 Table 14. To make it clearer, we also added the following paragraph (lines 422-425) to the manuscript:  "After IPTW weighting, the beneficiary and non-beneficiary groups had similar sociodemographic characteristics. The difference in proportions of each category between BFP beneficiaries and non-beneficiaries was lower than 10% (Table 1)." |
| 5. S4 Fig 3 does not match the data provided in S4 Table 3 - for example, the maximum propensity score in the non-BFP sample is stated as being 0.767 in the table, but is >0.8 in the figure. | Thank you for your comment. We recognized this inconsistency and have now aligned the data in the table with the figure. |
| 6. Please reduce the number of significant figures provided in the tables in S5 to 2. | Thank you for pointing this out. We have now reduced the number of figures to two as suggested. |
| 7. An intraclass correlation is provided in S6. What is the outcome that this is calculated for? Were multiple members of households hospitalised with psychiatric disorders? How many households were included, and what sizes were these? Is this referred to anywhere in the paper? I would suggest deleting this section. | We appreciate the opportunity to clarify this point. The outcome for which the ICC was calculated was overall mortality. The number of household members hospitalised with psychiatric disorders ranged from 1 to 4. The analysis included 69,192 households, with household sizes varying between 1 and 40 members. We ran intraclass correlation analyses to attend to a suggestion from Reviewer 3 to account for any potential cluster effects. However, we did not find any significant clustering. Therefore, we have now mentioned it in the manuscript "our sample had no clustering effect (S6 Text) "(lines 406-407) and transferred all the details to Appendix S6. But we are open to deleting all this information if you consider it is needed. |
| My comments have been addressed satisfactorily. I have no further comments. | Thank you. |

| **General editorial requests:** | **ANSWERS** |
| --- | --- |
| * We ask every co-author listed on the manuscript to fill in a contributing author statement, making sure to declare all competing interests. If any of the co-authors have not filled in the statement, we will remind them to do so when the paper is revised. If all statements are not completed in a timely fashion this could hold up the re-review process. If new competing interests are declared later in the revision process, this may also hold up the submission. Should there be a problem getting one of your co-authors to fill in a statement we will be in contact. Please do not add or remove authors without first discussing this with the handling editor. You can see our competing interests policy here: http://journals.plos.org/plosmedicine/s/competing-interests. | ok |
| * Please upload any figures associated with your paper as individual TIF or EPS files with 300dpi resolution at resubmission; please read our figure guidelines for more information on our requirements: http://journals.plos.org/plosmedicine/s/figures. While revising your submission, please upload your figure files to the PACE digital diagnostic tool, https://pacev2.apexcovantage.com/. PACE helps ensure that figures meet PLOS requirements. To use PACE, you must first register as a user. Then, login and navigate to the UPLOAD tab, where you will find detailed instructions on how to use the tool. If you encounter any issues or have any questions when using PACE, please email us at PLOSMedicine@plos.org. | Done |
| * Thank you for providing a data availability statement. We have a few requests for amendments here.  (1) Please note that a study author cannot be the contact person for the data. We ask that details be provided so that data requests can be made to a non-author institutional point of contact, such as a data access or ethics committee, as this helps guarantee long term stability and availability of data. Providing interested researchers with a durable point of contact ensures data will be accessible even if an author changes email addresses, institutions, or becomes unavailable to answer requests.  (2) We note that there are appears to be 3 datasets that have been accessed in this study. Could you provide access details for all three datasets (in the relevant metadata section) please? | We corrected the email for [cidacs.curadoria@fiocruz.br](mailto:cidacs.curadoria@fiocruz.br).  Regarding the 3 datasets, they were obtained from the CIDACS. There are restrictions on accessing the data. The data was licensed exclusively for use in this study and, due to privacy regulations from the Brazilian Ethics Committee, is not available to the public. Controlled access to the data may be granted upon reasonable request, provided that you obtain explicit permission from CIDACS (by emailing cidacs.curadoria@fiocruz.br) and receive approval from an ethics committee. |
| * We expect all researchers with submissions to PLOS in which author-generated code underpins the findings in the manuscript to make all author-generated code available without restrictions upon publication of the work. In cases where code is central to the manuscript, we may require the code to be made available as a condition of publication. Authors are responsible for ensuring that the code is reusable and well documented. Please make any custom code available, either as part of your data deposition or as a supplementary file. Please add a sentence to your data availability statement regarding any code used in the study, e.g. "The code used in the analysis is available from Github [URL] and archived in Zenodo [DOI link]" Please review our guidelines at https://journals.plos.org/plosmedicine/s/materials-software-and-code-sharing and ensure that your code is shared in a way that follows best practice and facilitates reproducibility and reuse. Because Github depositions can be readily changed or deleted, we encourage you to make a permanent DOI'd copy (e.g. in Zenodo) and provide the URL. | Done.  We included in our data avalilability statement:  "The code used in the analysis is available from Github [https://github.com/profacamilabonfim/Codes-from-the-paper.git] and archived in Zenodo [10.5281/zenodo.13750552]" |
| * We note that the datasets referred to in your study have been used by other publications. Nevertheless, it might be useful for readers if you could perhaps provide a short summary of the datasets in the Supporting Information files? This may enable the reader to understand the nature of the data a bit better without having to leave the article page. | The 100 Million Brazilian Cohort has been used in several studies; however, this is the first time we have focused on a specific vulnerable population—those who have been hospitalized due to a psychiatric disorder within this large cohort. We have added further information about the datasets in the Appendix S11 Text. |
| * Thank you for including the STROBE checklist in your Supporting Information. Could you kindly revise this so that you only use section and paragraph numbers, rather than page numbers? This is because page numbers can change at a later point in the publication process. Please also add the following statement, or similar, to the Methods: "This study is reported as per STROBE guideline (S1 Checklist)." | Done. We added the information in lines 234-235. |
| SUPPLEMENTARY MATERIAL  * Please cite your Supporting Information as outlined here: https://journals.plos.org/plosmedicine/s/supporting/information | Done. |
| REFERENCES  * Where website addresses are cited, please include the complete URL and specify the date of access (e.g. [accessed: 12/06/2023]). | Done |
